# Conformations of Macrocyclic Peptides Sampled by Nuclear Magnetic Resonance: Models for Cell-Permeability

**DOI:** 10.1021/jacs.3c09367

**Published:** 2023-12-08

**Authors:** Simon H. Rüdisser, Emmanuel Matabaro, Lukas Sonderegger, Peter Güntert, Markus Künzler, Alvar D. Gossert

**Affiliations:** † Department of Biology, ETH Zürich, Zürich 8093, Switzerland; ‡ Department of Chemistry and Applied Biosciences, ETH Zürich, Zürich 8093, Switzerland; § Institute of Biophysical Chemistry, Goethe University, Frankfurt am Main 60438, Germany; ∥ Department of Chemistry, Tokyo Metropolitan University, Hachioji, Tokyo 192-0397, Japan

## Abstract

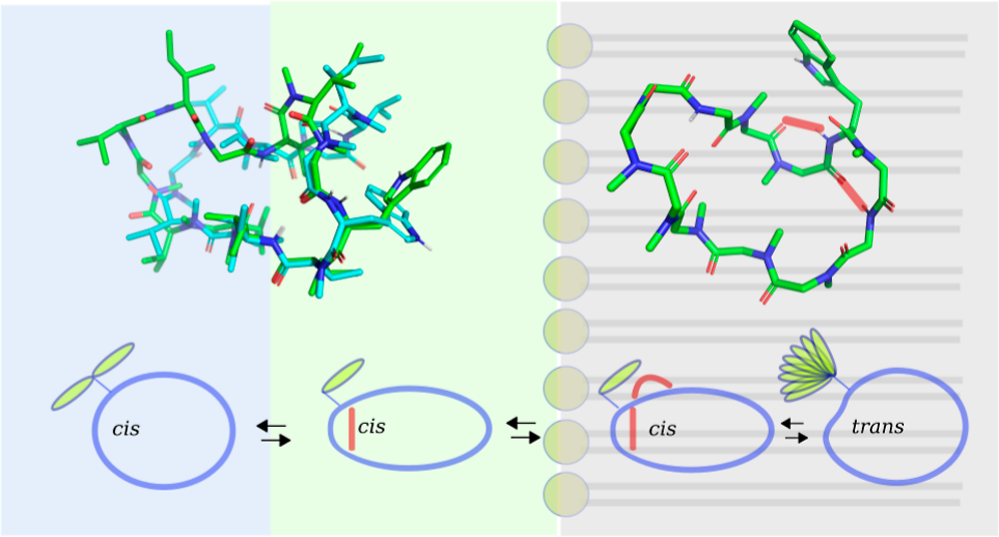

The biological activities and pharmacological properties of peptides and peptide mimetics are determined by their conformational states. Therefore, a detailed understanding of the conformational landscape is crucial for rational drug design. Nuclear magnetic resonance (NMR) is the only method for structure determination in solution. However, it remains challenging to determine the structures of peptides using NMR because of very weak nuclear Overhauser effects (NOEs), the semiquantitative nature of the rotating frame Overhauser effect (ROE), and the low number of NOEs/ROEs in *N*-methylated peptides. In this study, we introduce a new approach to investigating the structures of modified macrocyclic peptides. We utilize exact NOEs (eNOEs) in viscous solvent mixtures to replicate various cellular environments. eNOEs provide detailed structural information for highly dynamic modified peptides. Structures of high precision were obtained for cyclosporin A, with a backbone atom rmsd of 0.10 Å. Distinct conformational states in different environments were identified for omphalotin A (OmphA), a fungal nematotoxic and multiple backbone *N*-methylated macrocyclic peptides. A model for cell-permeation is presented for OmphA, based on its structures in polar, apolar, and mixed polarity solvents. During the transition from a polar to an apolar environment, OmphA undergoes a rearrangement of its H-bonding network, accompanied by a *cis* to *trans* isomerization of the ω torsion angle within a type VIa β-turn. We hypothesize that the kinetics of these conformational transitions play a crucial role in determining the membrane-permeation capabilities of OmphA.

## Introduction

Bioavailability, in combination with a high affinity for the target, is the key determinant of drug efficacy. For backbone *N*-methylated peptides, these properties are determined by the conformational states and the transition between states that is conformational dynamics.

Preorganization of the binding-competent state in solution facilitates binding to the target by conformational selection.^1,2^ Rapid exchange between hydrophobic and hydrophilic states is required for passive membrane diffusion, which is the most desirable mechanism for membrane permeation.^3^ These properties have been optimized by nature for naturally occurring peptide cyclization and *N*-methylation, for instance. The challenge is to confer synthetic peptide drugs with the desired properties.

Peptide drugs show, in general, low cell permeability due to the presence of backbone H-bond donors and acceptors. For these molecules, cell penetration can be improved by backbone *N*-methylation, which, however, reduces aqueous solubility and can interfere with target binding.^4^ Understanding conformations and their dynamics is key to rational drug design.^5^

Nuclear magnetic resonance (NMR) spectroscopy is currently the only method for studying conformation and dynamics at atomic resolution in solution. However, several problems limit the applicability of NMR for this purpose. The main sources of structural information in NMR, the nuclear Overhauser effect (NOE) and the rotating frame Overhauser effect (ROE), are sparse due to conformational dynamics and the absence of secondary structural elements in modified peptides. Although the ROE is, in principle, quantitative, experimental restrictions limit its application for precise structure determination. Specifically, suppression of signals due to J-coupling, which leads to total correlation cross-peaks, as well as the removal of the offset dependence of the ROE, are required. Various ROE experiments have been proposed to facilitate this quantitative analysis, with the EASY ROESY experiment^6^ being particularly noteworthy. These ROE experiments may still contain residual artifacts if not meticulously set up.

The NOE is, in principle, quantitative; however, for small molecules, the NOEs are typically weak due to fast molecular tumbling. The NOE effect is strong for slowly tumbling molecules, such as proteins with a molecular mass exceeding 10 kDa. For small molecules, e.g., macrocyclic peptides, strong NOE effects can be achieved by employing high-viscosity solvents^7^ and by reducing the temperature of the sample.^8^ The choice of solvent does not only affect the viscosity but also strongly influences conformation and dynamics. Solvent properties like polarity, dielectric constant, and the presence of hydrogen bond acceptors need to be considered, especially when studying modified macrocyclic peptides. Therefore, different solvents allow the investigation of molecules in diverse environments, enabling the deduction of properties relevant to target binding and cell-permeability.^9^ Solvent selection is of great importance for biological relevance. In this study, we utilized mixtures of CDCl_3_/*n*-hexadecane-*d*_34_, CD_3_OD/H_2_O, and DMSO-*d*_6_/H_2_O to emulate the apolar cell-membrane, the cytosol, and the binding epitope.

For proteins and nucleic acids, NMR structure determination protocols that rely on the semiquantitative analysis of NOE data are well established. NOE data are usually acquired at a single NOE mixing time and converted into different categories of upper distance restraints for the structure calculation. For molecules with a high density of restraints, *e.g.*, folded proteins, high-precision structures can be obtained despite the lack of precise upper and lower distance bounds.

Quantitative analysis of NOE data has been described for early structure determination work for proteins,^10,11^ as well as for DNA in combination with relaxation matrix analysis.^12,13^ In recent years, the exact NOE (eNOE) method, which allows for the determination of accurate distance information from NOE buildup data, has been re-established for proteins^14^ and nucleic acids.^15^ The eNOE method increases the information content of NMR restraints and is especially well-suited for molecules from which only a limited number of distance restraints can be obtained, as demonstrated in this study. The quantitative nature of the eNOE approach is particularly suitable for exploring fast-exchanging conformations, like in *N*-methylated peptide macrocycles, by treating the distance restraints as an average over multiple states.^14^

Biomolecules in solution undergo conformational exchange on the second to nanoseconds time scale.^16^ Dynamics are essential for biomolecular function, and an understanding of molecular motion is a prerequisite for rationalizing the mechanism of biomolecules. *N*-methylated peptides adopt multiple conformational states due to the lower number of intramolecular H-bonds compared to that of the unmethylated molecule. The transition between these states can be “fast” on the NMR time scale if the process involves crossing a low free energy barrier or “slow”, with a *k*_ex_ < 10 s^–1^ if a high energy barrier transition is involved. Fast crossing results in highly dynamic conformational ensembles, which are notoriously difficult to characterize. Both NOE and ROE experiments are sensitive to distance fluctuations at the nanosecond time scale, and various quantitative methods have been proposed to extract distances from these experiments.^17−19^ In order to characterize multiple conformations and the kinetics of conformational exchange, the kinetic ensemble approach was introduced recently.^20^ With the kinetic ensemble method, ensembles of structures and the interconversion rates between these sets of ensembles are calculated from the NOE data. Recent NMR relaxation experiments have provided compelling evidence for the presence of pervasive dynamics in proteins,^21^ specifically on the picosecond to nanosecond time scale with 1Å amplitude for proton–proton distance fluctuations.

**Figure 1:**
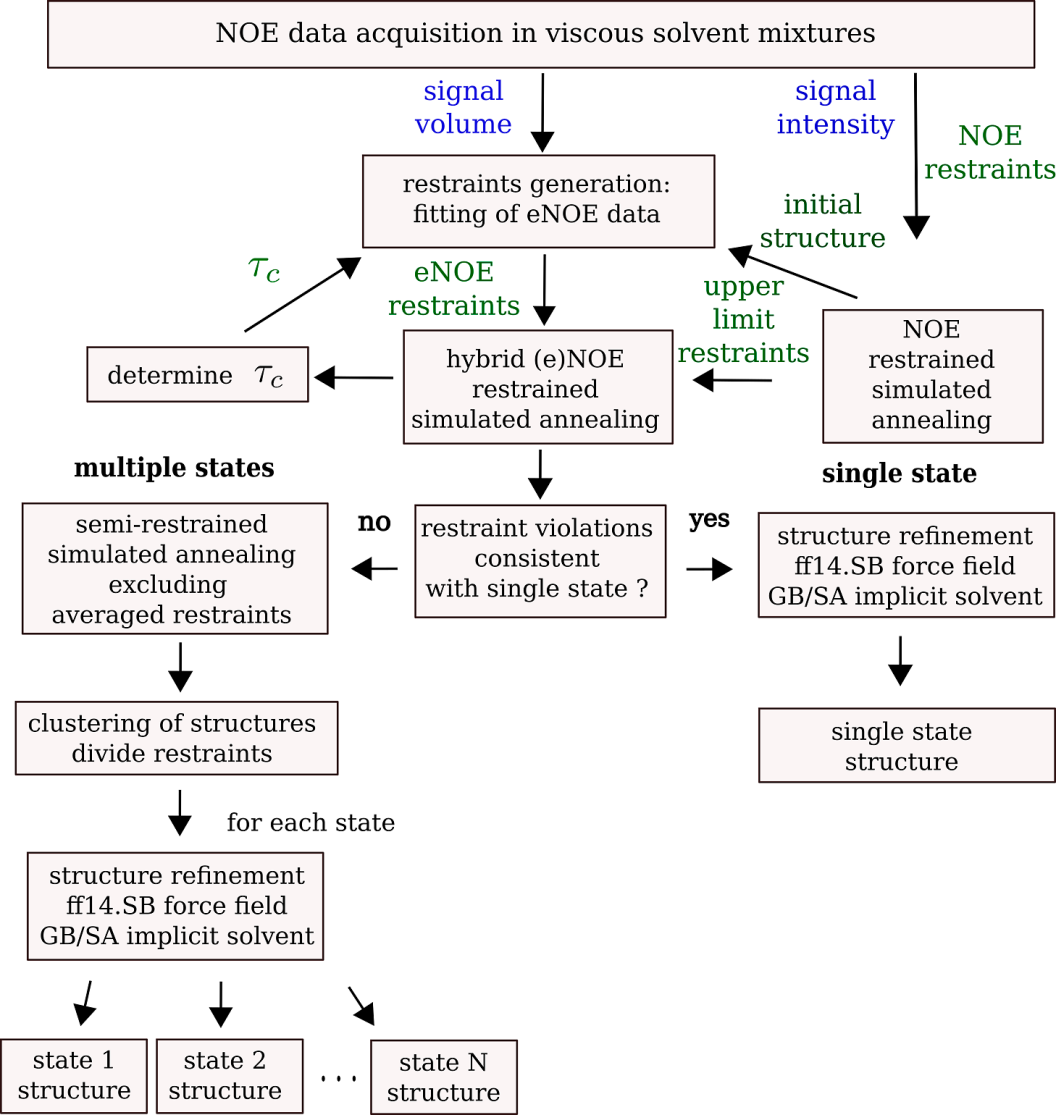
Workflow for structure determination utilizing hybrid (e)NOEs for modified macrocyclic peptides in viscous solvent mixtures. NOE buildup data are acquired in polar, apolar, and mixed polarity solvents. The rotational correlation time, τ_c_, is determined as described in the main text. Initial structures for spin-diffusion correction are calculated using semiquantitative NOE restraints. eNOE restraints are generated by fitting the buildup and decay NOE data and are thereafter combined with the NOE restraints to obtain hybrid (e)NOE restraints. Should the observed restraint violations in the resulting structures indicate conformational averaging, the corresponding restraints are partitioned into *N* states. Restrained simulated annealing has been performed utilizing CYANA.^52^ For each individual state, structures are refined by employing the ff14SB^55^ force field and the GB/SA implicit solvent model.^54^

In our work, we extend the eNOE method to study the conformations and dynamics of macrocyclic drugs with NMR by introducing a range of methodological improvements. First, high-viscosity solvent mixtures, polar, apolar, and polar/apolar, are used to mimic the different cellular compartments. Second, the eNOE method, which was previously described for proteins and nucleic acids, is adapted to small molecules. Third, the structure calculation protocol is modified for flexible macrocycles with low NOE density. We show that our method improves the precision of structure determination considerably, and, most importantly, it allows one to obtain structures of dynamic macrocycles with a limited set of eNOE restraints. The workflow that describes our approach is shown in Figure 1.

The method has been applied to two peptide natural products, cyclosporin A (CsA) and the fungal nematotoxic peptide omphalotin A (OmphA).

The cyclo-undecapeptide CsA is nonribosomally produced by the fungus*Tolypocladium inflatum*and is used as an immunosuppressant in organ transplantations.^22^ Seven of the backbone amide nitrogens are methylated. The structure of CsA in its free state, in hydrophobic and hydrophilic environments, and when bound to its protein target, cyclophilin, is a major focus of research for rationalizing its oral availability.^8,23−26^ In the bound state, the structure of CsA closely resembles the structures in the aqueous environment, whereas the structures in lipophilic solvents and the crystal^23^ reveal a considerably different conformation. The binding of CsA to cyclophilin involves a *cis* to *trans* isomerization of the Mle 9–Mle 10 peptide bond, resulting in a slow onset of inhibition.^27^

The primary mechanism of cell membrane permeation for CsA is passive diffusion.^28^ This process is facilitated by the chameleonicity of CsA, which is characterized by its change of conformation from “open”, present in water, to a “closed” conformation that forms intramolecular H-bonds while exposing the lipophilic side chains to the exterior.^29^ Other transport mechanisms may also contribute to the oral availability of CsA. Studies have shown that, for *N*-methylated cyclic hexapeptides, the number and position of *N*-methylated amide groups do not correlate with intestinal permeability.^30^ These findings suggest that transport through the tight junction of the intestine wall and active transport via peptide transporters, *e.g.*, PepT1,^31^ also contribute to the oral availability of cyclic peptide drugs.

The role of conformational flexibility and the rates of interconversion between states for membrane permeability have been highlighted for CsA using molecular dynamics simulations.^3,32^ A recent study employing a combination of X-ray and single-crystal neutron diffraction has revealed additional conformations of CsA in water/methanol mixtures.^33^

The second peptide studied in this work is OmphA, a much less investigated natural product. OmphA is a nematotoxic cyclic peptide with 9 of the 12 backbone nitrogens methylated.^34^ It is produced by the fungus*Omphalotus olearius*through the autocatalytic activity of an α-*N*-methyltransferase fused to its peptide substrate.^35^ Based on our studies, we propose a model for cell permeation and a hypothesis for the binding-competent state of OmphA.

## Theory

Here, we briefly recapitulate the relevant equations that are required for the analysis of NOE data in general and specifically for the application to macrocyclic peptides in viscous solvents. The NOE effect, or cross-relaxation, is described for a two-spin system with and without internal dynamics. Internal dynamics due to conformational exchange in the millisecond or submillisecond time scale lead to population-averaged NOE distance restraints. Subsequently, the incorporation of averaged eNOE restraints into the structure calculation is described.

### Cross-Relaxation

Cross-relaxation due to dipolar interactions, the NOE, is the main source of spatial information for structure determination from NMR data.^36^ The cross-relaxation rate σ_*ij*_ between two nuclei *i* and *j*, typically ^1^H in biomolecules
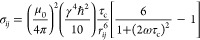
1is dependent on the distance *r*_*ij*_ between the two nuclei and the correlation time τ_c_ that describes the rate of isotropic tumbling of the vector connecting the two nuclei. The constants in eq 1 have their usual meaning: μ_0_ = 1.25663706143 × 10^–6^ N A^–2^ is the magnetic constant, γ = 42.576 × 10^6^ s^–1^ T^–1^ is the gyromagnetic ratio of ^1^H, and *ℏ* is the reduced Planck constant. The field strength for protons, ω = γ*B*_o_, is given in rad s^–1^, where *B*_o_ is the magnetic field strength in Tesla.

For rigid and spherical molecules, τ_c_ is identical to the overall isotropic rotational diffusion correlation time τ_overall_. The fast internal motion described by an internal correlation time τ_int_ ≪ τ_overall_ reduces the overall cross-relaxation rate according to eq 2.
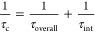
2

Let us investigate the example of an NOE transfer from spin *i* to spin *j* in an isolated spin-pair. The Solomon equations^37^ (eq 3)
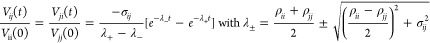
3describe the buildup and decay of cross-peak volumes *V*(*t*) in terms of the cross-relaxation rate σ_*ij*_ and the autorelaxation rates ρ_*ii*_ and ρ_*jj*_.

*V*_*ii*_(*t*) and *V*_*jj*_(*t*) are the volumes of the source and destination signals at the NOE mixing time *t*, respectively. The magnetization at destination proton *j*, which is proportional to *V*_*ij*_(*t*), is normalized by *V*_*ii*_(*t*). The autorelaxation rate ρ_*ii*_ and the signal volume for the source proton at *t* = 0, *V*_*ii*_(0), are determined by fitting an exponential decay function, *V*(*t*) = *V*(0)*e*^–ρ*t*^, to the diagonal peak volumes at the different mixing times. Source and destination signals can be affected differently by chemical exchange, which decreases the signal intensity while preserving its volume. Note that σ_*ij*_ = σ_*ji*_ for symmetry reasons.

In order to calculate τ_c_, an intramolecular distance between two protons with a fixed covalent geometry can be used as a reference. For example, the distance between the two geminal protons H^α2^ and H^α3^ in Gly or between H^δ^ and H^ϵ^ in the aromatic ring of Tyr or Phe residues can be used for calibration.
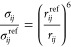
4

Equation 4 describes the relationship between two distances and the corresponding cross-relaxation rates. In practice, we back-calculated τ_c_ from several reference distances and used the resulting average τ_c_ in the subsequent computation of distance restraints.

### Multiple Conformations in Fast Exchange

The NOE reports on time-averaged distances of conformations that exchange during the NOE mixing period. For the calculation of the average NOE, ensemble-averaging of Boltzmann-weighted structures can be used.^38^ Alternatively, time-averaging of structures from the time course of an MD simulation can be applied.^39,40^

For an ensemble of *N* structures with respective populations *p*_*n*_, the average distance can be expressed as shown in eq 5.
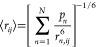
5

Because of the *r*^–6^-dependence of the NOE effect, shorter distances have a much stronger contribution to the ensemble averaged NOE.^41^ Note that .

The population *p*_*n*_ can be assessed from NOE distances by complete cross-validation.^42^ Alternatively, *p*_*n*_ can be obtained from algorithms that simultaneously refine multiple copies of the structure and adjust the weights of the individual conformers in such a way that the restraint energy is minimized.^43^ More recently, structural correlations have been used to estimate the populations of states.^44^

In general, an accurate estimation of *p*_*n*_ requires many and precise NOE restraints. For this reason, no attempt to estimate *p*_*n*_ has been made here, and we set *p*_*n*_ = 1/*N* for an *N*-state model.

The *N* conformations, or states, that contribute to the averaged NOE need to be calculated based on force fields that accurately describe the free energy of these conformations. The absence of restraints that correspond to a single state necessitates an accurate description of the molecular forces of the ensemble averaged states.

## Experimental Section

### Production of OmphA and OmphA Trp1–Ala1

OmphA was produced from the*Pichia pastoris*strain expressing the *ophMA* and *ophP* that were previously reported,^45^ while the OmphA Trp1–Ala1 variant was produced from a*P. pastoris*(SMA2903) expressing *ophP*, which was transformed with a plasmid carrying *ophMA* W400A. To produce the two peptides, the method described by Matabaro *et al*. was applied.^46^ Briefly, cells were first precultured in buffered glycerol-containing complex medium (BMGY) until the OD_600_ reached between 2.0 and 6.0. After centrifugation at 2000 rpm at room temperature, cell pellets were washed twice with a methanol-containing complex medium (BMMY). The peptide production was induced by maintaining the cells (1.0 as a starting OD_600_) for three days in BMMY with a 0.5% methanol (v/v) spike every 24 h. After harvesting cells by centrifugation, cells were resuspended in 2× PBS containing 0.5 mm diameter glass beads and lysed by a planetary mill (Pulverisette 7, Fritsch, Germany) at level 3,400 rpm and 3 repetitions. After centrifugation at 8000 *g* for 30 min, the cell lysate supernatant was collected and used for liquid–liquid extraction. The liquid–liquid extraction was performed by mixing an equal volume of cell lysate supernatant with ethyl acetate (1:1, v/v) in a separatory funnel. After vigorous mixing and resting for a few minutes, the upper organic phases were collected and evaporated using a rotary evaporator. The dried extracts were redissolved in methanol and purified on a C18 column (Phenomenex Luna 5 μ C18, 10 × 250 mm) with reversed-phase HPLC, with UV set at λ = 210 nm and λ = 280 nm. Further details on the HPLC purification method are described in Matabaro et al.^46^ The fractions corresponding to the peptides were collected and evaporated. The resulting dried pellets were weighed and kept at −20 °C until use.

### NMR Experiments

CsA (Sigma, 30024-25MG) has been dissolved in a mixture of 60 μL of hexadecane-*d*_34_ (98 atom %, Aldrich 489603-100MG) and 120 μL CDCl_3_ (99.8 atom %, Aldrich 151823-0.75ML). The total sample volume was 140 μL, which resulted in a CsA concentration of 23 mM. The solution was transferred to a 3 mm NMR tube (Norell, S-3-HT-7), and NMR data were acquired at 274 K at a 700 MHz Bruker AVNEO NMR spectrometer equipped with a CP-TCI-H-C/N-D 05 Z probe head. OmphA has been dissolved in a mixture of 130 μL of hexadecane-*d*_34_ (98 atom %, Aldrich 489603-100MG) and 500 μL of CDCl_3_ (99.8 atom %, Aldrich 151823-0.75ML). The concentration of OmphA was 20 μM and was determined by comparing the intensities of the *N*-methyl signals to those of a reference sample containing CsA. NMR data were acquired at 278 K in 5 mm NMR tubes. For the sample in polar solvents, OmphA was dissolved in 100 μL of CD_3_OH (99.96%, Sigma 44758-0.25ML) and 40 μL of deionized water. The solution was transferred to a 3 mm DMSO-*d*_6_ matched Shigemi NMR microtube (Sigma, Z569739-1EA). The sample in DMSO-*d*_6_/H_2_O was prepared in 450 μL of DMSO-*d*_6_ (99.5 atom %, Armar 015000.2050) and 50 μL of deionized water and transferred to a 5 mm NMR tube. Data were acquired on a Bruker AVANCEII 750 MHz spectrometer equipped with a PA-TXI H-C/N-D 05 Z probe head. The W1A variant of OmphA was dissolved in 70 μL CD_3_OH (99.96%, Sigma 44758-0.25ML) and 30 μL deionized water. The solution was transferred to a 3 mm DMSO-*d*_6_ matched Shigemi NMR microtube (Sigma, Z569739-1EA). The sample in apolar solvents was prepared by dissolving the W1A variant in 130 μL CDCl_3_ (99.8 atom %, Aldrich 151823-0.75ML) and transferring the sample to a CDCl_3_ matched Shigemi NMR microtube (Sigma, Z569720-1AE). 2D NOESY experiments with zero-quantum filtering^47^ (Bruker pulse sequence: noesygpphzs) at various mixing times were acquired on a Bruker AVNEO 700 MHz (CsA) and AvanceIIIHD 900 MHz (OmphA) spectrometer equipped with a CP-TCI H-C/N-D 05 Z probe head. NOESY spectra for CsA have been acquired with a recycling delay (prescan delay) of 1.5 and 10.0 s to investigate its influence on auto- and cross-relaxation rates. All other NMR experiments were performed with a recycling delay of 1.5 s, including the NOE experiments for OmphA. The NOE mixing times are as follows: For CsA: 60, 100, 150, 200, 250, 300, 350, 400, 450, 500 ms; for OmphA in CDCl_3_/*n*-hexadecane-*d*_34_: 100, 200, 300, 400 ms; for OmphA in CD_3_OD/H_2_O: 100, 200, 300, 400 ms. The time needed to acquire the NOESY data is as follows: approximately 3 h for 23 mM CsA, utilizing a prescan delay of 1.5 s, a NOE mixing time of 500 ms, and 8 increments. Approximately 15 h for ca. 20 μM OmphA with the prescan delay set to 1.5 s, a NOE mixing time of 500 ms, and 32 increments.

2D NOESY experiments were acquired with 2048 complex data points in the direct dimension and 256 data points in the indirect dimension. For resonance assignment, standard HSQC (hsqcetgp), HMBC (hmbcgpplpndqg), and TOCSY (dipsietgp) were applied. The pulse sequences from the Bruker library are given in brackets. Assignments for peptides have been achieved as described in the literature.^8^ For the samples in CD_3_OH/H_2_O, the solvent signals were suppressed by applying presaturation or the watergate sequence with soft selective pulses.^48^ The residual CHCl_3_ resonance at 7.28 ppm from the deuterated solvent or the H_2_O signal at 5.04 ppm was used to reference the ^1^H spectrum.

### NMR Data Processing

The FID was multiplied with a π/2-shifted squared sine function and zero-filled to 4096 data points for CsA and 2048 data points for OmphA before the Fourier transformation. Data acquisition and processing were done with TopSpin 4.0.7 (Bruker Biospin). Peak picking and spectral analysis for the assignment were done using CCPNmr 2.5.^49^ Peak volumes in 2D NOESY spectra were determined using the program seriestab.tcl of the nmrPipe system.^50^ NOE decay and buildup rates were fitted using the eNOE module^51^ in CYANA 3.98.15^52^ using corrections for spin diffusion.

### Structure Calculation

Cross-relaxation rates were converted to distance restraints according to eq 1 using the eNORA^51^ module in CYANA 3.98.15.^52^ The following values for τ_c_ were applied: τ_c_ = 0.47 ns for CsA, 0.40 ns for OmphA in CDCl_3_/*n*-hexadecane-*d*_34_, and 0.46 ns in CD_3_OH/H_2_O. Corrections for spin diffusion were applied to the eNOE data.^53^

The initial structures for spin diffusion correction were computed by restrained simulated annealing with semiquantitative NOE restraints (noeassign macro in CYANA 3.98.15^52^). Seven cycles of simulated annealing with 1000 steps were performed, and the 20 structures with the lowest CYANA target function (restraint violation penalties) were selected.

Semiquantitative and eNOE restraints were combined and applied during restrained simulated annealing (structcalc macro in CYANA 3.98.15^52^). Hybrid (e)NOE restraints were applied without any internal scaling or recalibration in CYANA. For OmphA, 500 structures were calculated (50,000 steps), and the 100 structures with the lowest CYANA target function were selected. For CsA, 100 structures were calculated (50,000 steps), and the top 20 structures were selected. The selected structures were subsequently refined using the sander module from AmberTools22.^54^ The ff14SB^55^ force field was used for restrained energy minimization and subsequent restrained simulated annealing utilizing sander. The solvent was implicitly considered by GBSA (generalized Born/surface area) simulations with an external dielectric constant of 3.9 for CDCl_3_/*n*-hexadecane-*d*_34_ or 78.5 for CD_3_OD/H_2_O. For both simulated annealing using CYANA and structure refinement using AmberTools, *r*^–6^ averaging of distances was applied. H-bond restraints were applied during restrained simulated annealing and minimization by applying upper-limit restraints of 2.0 Å between the donor and the acceptor and 3.0 Å between the acceptor and the heavy atom attached to the donor atom. The treatment of H-bonding restraints mirrors that of (e)NOE restraints: a distance-dependent restraint violation term is calculated and incorporated into the total restraint violation penalty. Both H-bonding and (e)NOE restraints are given equal weight. The peptide bond angle ω in *N*-methylated aminoacids was kept free to switch between *cis* and *trans* during restrained simulated annealing.

Minimization for structure refinement using sander was done with a maximum of 1000 cycles. The parameters for simulated annealing for refinement are as follows: heating to 500 K, equilibrating at 500 K, slow cooling to 0 K, and slowly ramping up the distance and torsion angle restraints.

## Results and Discussion

### High-Viscosity Solvents as Mimics for Different Cellular Environments

The magnitude and sign of the NOE, which is the primary source of structural information in NMR, depend on the rotational correlation time, τ_c_, of the molecule. The NOE varies between 0.5 for rapid tumbling and −1 for slowly tumbling molecules. In the intermediate tumbling regime, the NOE effect vanishes. Molecules with a molecular mass of about 1000 Da, which is typical for small peptides, are in the intermediate tumbling regime, which results in a very weak NOE. Therefore, to reduce the tumbling rate and to observe more pronounced NOEs for peptides, high-viscosity solvents and low temperatures are required.^7^ An illustrative example for NMR structure determination of CsA at low temperatures can be found in the work of Kessler *et al.*,^8^ where the structure of CsA was determined in CDCl_3_ at 252.5 K.

The solvent strongly influences the conformation and dynamics of the macrocyclic peptides. As a surrogate for the apolar environment within the cell membrane, we employed a mixture of CDCl_3_ and *n*-hexadecane-*d*_34_. While CDCl_3_ serves mainly as a cosolvent, *n*-hexadecane-*d*_34_ is chemically similar to the hydrocarbon tail of phospholipids. Partition coefficients between water and *n*-hexadecane are routinely measured to predict passive permeability in early drug discovery.^56,57^ For the simulation of the cytosolic environment, we utilized a mixture of H_2_O and CD_3_OH. CD_3_OH is essential to increasing the viscosity and to dissolve *N*-methylated macrocyclic peptides that are often water-insoluble. To mimic the binding epitope for macrocyclic peptides, we utilized a mixture of DMSO-*d*_6_ and H_2_O. This solvent provides hydrogen bond acceptors and donors, as well as hydrophobic contacts, which are commonly observed in peptide–protein binding interfaces.^58^

The high-viscosity solvent mixtures used to simulate various cellular environments are listed in Table 1.

**Table 1:** High-Viscosity Solvent Mixtures Are Employed to Mimic Cellular Environments^a^

solvent mixture	v/v (%/%)	τ_c_/ns (*T* = 279 K)	η/*cP*
apolar:	70/30	0.47 (CsA),	
CDCl_3_/*n*-hexadecane-*d*_34_		0.40 (OmphA)	
mixed polarity:	90/10	≈2.2 from S–E	≈4^60^
DMSO-*d*_6_/H_2_O			
polar:	60/40	0.46 (OmphA),	≈1.6^61^
CD_3_OH/H_2_O		≈0.9 from S–E	

a The determination of the τ_c_ values followed the described procedure. Additionally, for comparison, τ_c_ values were calculated for the specified viscosity using the Stokes–Einstein (S–E) law (equation 6.2 from Wüthrich 1986^59^). The radius of an equivalent sphere for CsA and OmphA was estimated to be 8 Å based on the dimensions of CsA.

### Determination of τ_c_ for Peptides without Isotope Enrichment

The rotational correlation time for proteins is typically calculated from ^15^N–{^1^H} NOE and ^15^N *T*_1_/*T*_2_ or ^13^C–{^1^H} NOE and ^13^C *T*_1_/*T*_2_ relaxation data.^62^ This approach requires isotopic enrichment with ^15^N or ^13^C, which is often not feasible for modified macrocyclic peptides. ^13^C relaxation and ^13^C–{^1^H} NOE data acquired at natural abundance can also be used to calculate τ_c_.^63^ However, measurements at natural abundance are time-consuming and insensitive and require substantial amounts of the difficult to prepare natural product.

As an alternative, we have calculated τ_c_ by referencing to an intramolecular distance between two protons with fixed covalent geometry. For example, the distance between the two geminal protons H^α2^ and H^α3^ in Gly or H^δ^ and H^ϵ^ and in the aromatic ring of Tyr or Phe residues can be used for calibration.

**Figure 2:**
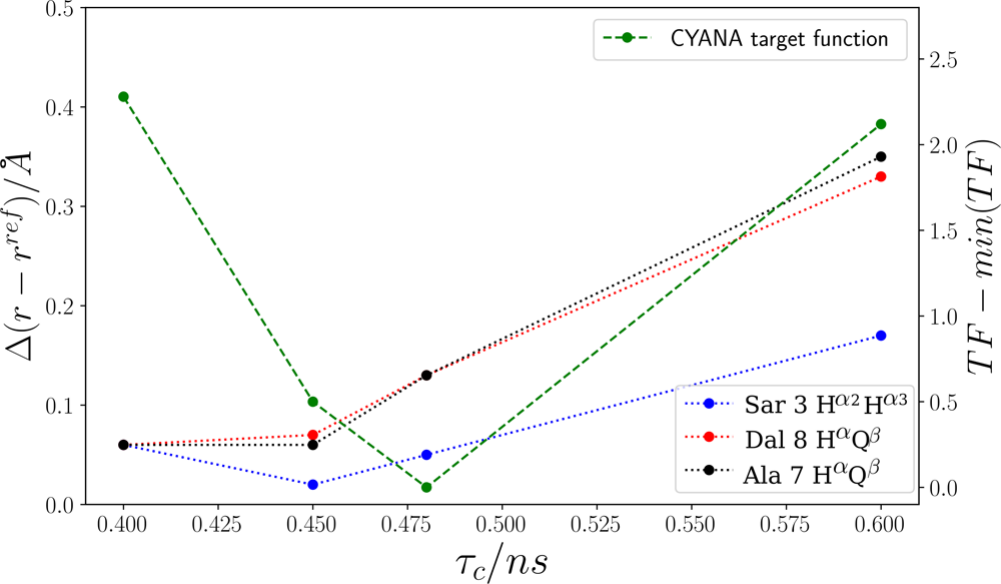
Average CYANA target function for the 20 top-ranked structures has been calculated as a function of τ_c_ for CsA. For comparison, the differences between the reference and calculated distances for Sar 3 H^α2^, H^α3^, and Ala 7/Dal 8 H^α^, Q^β^ are shown. The reference distance for the geminal protons in Sar 3 is 1.72 Å for H^α2^, H^α3^, and 3.0 Å for Ala 7/Dal 8 H^α^, Q^β^. Note that Q^β^ is a pseudoatom that represents the chemically equivalent methyl protons. In total, 163 hybrid (e)NOE restraints have been applied.

If the reference distance includes methyl resonances, *r*^–6^ averaging is used for the three protons in CYANA.^64^ This results in a slight underestimation of distances.^65^Figure 2 displays the results of referencing distances to individual protons for Sar 3 and distances between individual protons and methyl groups for Ala 7 and Dal 8. Both approaches result in the same minimum value for τ_c_. Therefore, we consider the error arising from the *r*^–6^ averaging to be negligible.

One drawback of referencing intramolecular distances is the difficulty in identifying spin pairs with fixed distances and with resolved resonances in the NOESY spectrum. In such a case, τ_c_ can be obtained by minimizing the sum of all restraint violations, including distances that are not fixed by covalent geometry. For this approach, distance restraints are calculated from a range of τ_c_ values, and a series of structures is computed in CYANA by restrained simulated annealing. Figure 2 shows the corresponding values for CsA. The value corresponding to the structure with the lowest CYANA target function is subsequently used for eNOE calculations. For the test case CsA, the τ_c_ value of 0.47 ns obtained from the target function minimizing approach is close to the value reported from ^13^C relaxation and ^13^C–{^1^H} NOE data for CsA.^63^ The τ_c_ value determined here accounts for overall molecular tumbling as well as internal motions and may, therefore, be described as an effective τ_c_. It is worth noting that, according to eq 1, an error in τ_c_ of 10% results in a distance error of only 1%.

We note that internal referencing for the determination of τ_c_ has a few pitfalls.^66^ (i) Due to internal motion, the τ_c_ values may be different for different pairs of protons. (ii) Cross relaxation to a third spatially close proton (spin diffusion) leads to deviations from rates for idealized two-spin approximation. (iii) At short NOE mixing times, zero-quantum interference distorts NOE signals in 2D NOESY spectra, which in turn leads to erroneous signal intensities and volumes. We have addressed these issues as follows: (i) proton pairs within the mostly rigid peptide backbone were used as a reference. (ii) Spin-diffusion is considered by applying a full-relaxation-matrix calculation.^53^ (iii) Zero-quantum interference is eliminated by applying a frequency sweep pulse in combination with a gradient during the NOE mixing delay.^47^ Most importantly, by measuring peak volumes, positive and negative zero-quantum signals will cancel out.

For OmphA, the method of minimizing the CYANA target function was used since heteronuclear natural abundance spectra could not be acquired because of sample limitations, and no or only a few signals for referencing were resolved.

### Cross-Relaxation Rates from Buildup Data: Normalization, Integration, and Error Propagation

To determine the cross-relaxation rate σ, the cross-signal is normalized relative to the diagonal-signal. However, pairs of protons involved in a NOE transfer can experience different degrees of line broadening due to conformational exchange. For instance, the diagonal signal of the source proton H_*i*_ may undergo stronger line broadening compared to the signal of the receiving proton H_*j*_. While line broadening reduces signal intensities, the signal volumes remain unaffected and are therefore used for data fitting. Consequently, measuring peak volumes is the preferred method for obtaining cross-relaxation rates. However, due to signal overlap, measuring intensities is more practical in crowded protein spectra.^67^

**Figure 3:**
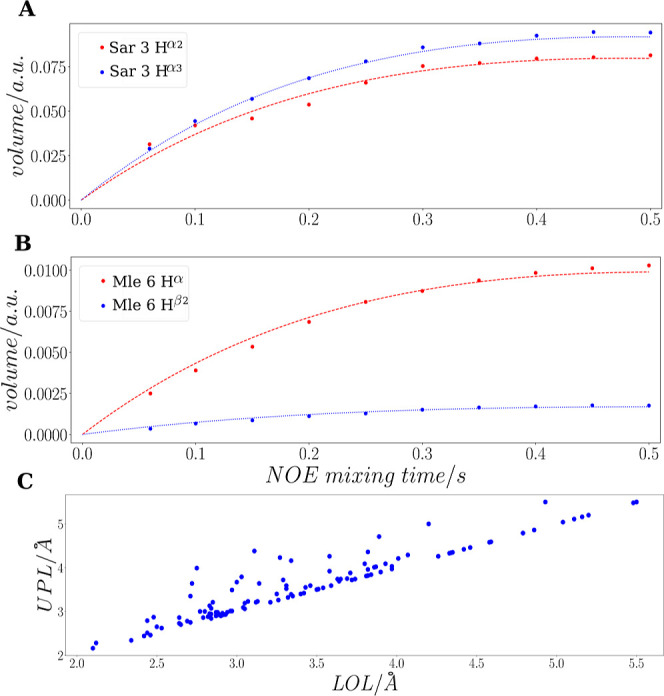
eNOE buildup data for the geminal protons H^α2^, H^α3^ of Sar 3 (A) and H^α^, H^β2^ of Mle 6 (B) (CsA in CDCl_3_/*n*-hexadecane-*d*_34_) are shown. The buildup (dotted lines) curves were fitted to the data according to eq 3. For Mle 6, the obtained restraints are *r*_*ij*_ = 3.61 Å and *r*_*ji*_ = 2.69 Å. Differing restraints are due to normalization to different diagonal-peak volumes, *V*_*ii*_ = 1.029 × 10^12^ (H^β2^, blue curve) and *V*_*jj*_ = 2.051 × 10^11^ (H^α^, red curve). *V*_*ii*_ is significantly larger than *V*_*jj*_ because of signal overlap, which cannot be resolved by deconvolution. In contrast, for Sar 3, *V*_*ii*_ = 7.087·10^10^ (H^α3^, blue curve) and *V*_*jj*_ = 7.340·10^10^ (H^α2^, red curve), which results in *r*_*ij*_ = 1.80 Å and *r*_*ji*_ = 1.76 Å. To take into account possible errors in volume determination, we have, therefore, set the upper and lower limit distance limits (UPL and LOL) to max(*r*_*ij*_, *r*_*ji*_) and min(*r*_*ij*_, *r*_*ji*_), respectively. The correlation time, τ_c_ = 0.47 ns, for restraint generation was determined as described in the main text. Deviations of the restraints *r* from the fixed distance between geminal protons, *r* = 1.72 Å, can be attributed to local variations in τ_c_. (C) Lower- and upper-limit distance restraints are shown before the correction is applied for methyl groups. The NOESY data have been acquired with a relaxation delay of 10 s to allow for complete relaxation prior to the next scan.

For peptides, the signal overlap is less severe, making the volume determination feasible. Nevertheless, determining the peak volume on the diagonal might still be challenging in 2D NOESY experiments due to signal overlap. Incorrect diagonal-peak volumes result in inaccurate normalization and significant discrepancies between σ_*ij*_ and σ_*ji*_, as shown in Figure 3.

The eNOE protocol for proteins^67^ and for nucleic acids^15^ utilizes average distance restraints derived from both cross-peak intensities, corresponding to cross-relaxation from H_*i*_ to H_*j*_ and from H_*j*_ to H_*i*_, in heteronuclearly edited 3D NOESY experiments to eliminate the influence of line broadening.^68^ Furthermore, averaging of bidirectional eNOE restraints cancels the difference in magnetization loss during the experiment due to, *e.g.*, INEPT transfer and water-suppression elements.

In cases where the signal intensity of strongly overlapped diagonal-signals cannot be determined, normalization to the mean diagonal-signal intensity for the corresponding proton type allows for the calculation of what is referred to as “generic normalized restraints” for proteins.^69^ However, for peptides with only a few protons of a given type, the statistics for computing mean intensities are poor and often not necessary due to resolved signals.

In our application, 2D NOESY experiments were performed in deuterated solvents. Differences between σ_*ij*_ and σ_*ji*_ are observed, as shown in Figure 3C. These differences originate primarily from the normalization of the diagonal-peak volume, low signal-to-noise, and cross-peak overlap. To explore the impact of the prescan relaxation delay on the cross-relaxation rates and, therefore, on the resulting distance restraints, NOESY data for CsA have been acquired with short and long delays. No significant differences were observed between eNOE restraints obtained with a prescan delay of 1.5 s in comparison to 10.0 s (Figure S1). To address other potential sources of errors, the data were analyzed as described in the following: the quality of the fit to the NOE decay and buildup data was inspected visually. Data for proton pairs with a low signal-to-noise ratio and/or poor fit were discarded. Since eNOE distance restraints could not be obtained for those proton pairs, they were treated as semiquantitative restraints. To take into account the remaining uncertainties in signal volume, the upper limit distance restraint is set to max(*r*_*ij*_, *r*_*ji*_) and the lower distance limit to min(*r*_*ij*_, *r*_*ji*_). Restrained simulated annealing was performed with combined eNOE and semiquantitative restraints by supplementing eNOE upper- and lower-limit restraints with semiquantitative upper-limit restraints. We named this approach the hybrid (e)NOE method.

### Structure Calculation Protocol for Macrocyclic Peptides by Simulated Annealing

The calculation of a structure from NOE data is a complex optimization problem. The target function to be optimized is a hybrid energy function with contributions from experimental restraint violations and force-field terms. Global optimization, *i.e.*, minimization of the target function by molecular dynamics-based restrained simulated annealing, is commonly used.^70^ Restrained simulated annealing by torsion angle dynamics is capable of efficiently calculating the structures of large proteins.^64^ Here, we used restrained simulated annealing by torsion angle dynamics to exhaustively sample the conformational space of peptides. It has been shown that conformational sampling by torsion angle dynamics, as implemented in CYANA, is superior to long-time molecular dynamics if slow motions are present.^71^

In the case of modified macrocyclic peptides, the restrained simulated annealing protocol in CYANA was specifically adjusted, as explained in the following. Modified macrocyclic peptides typically exhibit a low density of restraints due to the absence of secondary structural elements and increased dynamics. Consequently, the energy hypersurface becomes rugged, characterized by numerous local minima that can trap the minimizer. Additionally, the lack of experimental restraints results in slow convergence. Furthermore, the absence of a physical force field in CYANA leads to degeneracy among the minima in the potential energy hypersurface.

To address the issue of slow convergence, we have increased the number of torsion angle dynamics steps to 50,000. In order to thoroughly sample local minima, a total of 500 structures were calculated using restrained simulated annealing in CYANA. The subsequent refinement of structures is performed by employing the ff14SB^55^ force field with an implicit solvent.^54^ This allows us to discriminate between local minima that appear degenerate in the CYANA target function hypersurface. Our two-step approach, involving comprehensive sampling through restrained simulated annealing torsion angle dynamics and subsequent conformer selection using accurate force fields, yields precise structures, as will be evaluated further below.

### Incorporation of Conformation Averaged NOE Restraints into Structure Calculations

In this study, we propose a novel method for incorporating averaged eNOE restraints into structure calculations. Our approach takes advantage of efficient and extensive conformational sampling using restrained simulated annealing in CYANA by torsion angle dynamics. In addition, accurate force fields, in combination with an implicit solvent model, are applied to calculate the structures of the individual states.

The method consists of the following steps:

(i) Identification of averaged eNOE restraints, denoted as the set *C*, that are inconsistent with a single state. To achieve this, we perform restrained simulated annealing, including the full set of restraints. Averaged restraints result in violations for proton-pairs that are close in space.

(ii) Exhaustive conformation sampling is performed using restrained simulated annealing in CYANA. Averaged restraints *C* are excluded (semi-restrained) to allow unrestrained sampling for residues that adopt multiple states.

(iii) The resulting structures are thereafter grouped into *N* clusters. The objective of clustering is to identify distinct states based on the similarity of structures. Similarities are calculated from the ^1^H–^1^H distances of all of the structures in the bundle. Only ^1^H–^1^H distances that correspond to averaged restraints were considered. The distance metric for clustering is shown in eq 6.
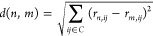
6

The sum in eq 6 encompasses all averaged restraints *C*, with *r*_*n*,*ij*_ and *r*_*m*,*ij*_ denoting the proton–proton distances corresponding to a violated distance restraint between atoms *i* and *j* in structures *n* and *m* within the bundle, respectively.

The *k*-medoids algorithm, which is implemented in the sklearn-learn^72^ Python module, is used for clustering. *k*-medoids clustering minimizes the sum of all distances within a cluster. The cluster medoid is the structure whose dissimilarity to all other cluster members is minimal.^73^

To visually assess the separation of clusters, pairs of distances are plotted. Alternatively, the adjusted mutual information, as described by Xuan Vinh *et al.*(74) can be utilized to evaluate the clustering quality. The *N cluster* medoids are representative structures for each respective cluster. Each of the *N* cluster medoids contributes to the averaged eNOE according to eq 5.

(iv) Splitting of averaged restraints from set *C* into *N* restraints for each cluster. For the purpose of individual structural refinement of the medoids, the averaged eNOE restraints are divided into *N* restraints, denoted as *r*_*n*,*ij*_^noe^ for protons H_*i*_ and H_*j*_, where *n* = 1, ···, *N*. The splitting of restraints is accomplished by minimizing the target function (TF), which is defined as the difference between ^1^H–^1^H distances in the cluster medoids, *r*_*n*,*ij*_, and the divided eNOE restraints, *r*_*n*,*ij*_^noe^, as well as the observed averaged eNOE, *r*_*ij*_^noe^, and the calculated average distance ⟨*r*_*ij*_⟩, as described in eq 7.
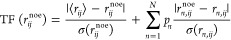
7

Upper- and lower-limit restraints are split independently. Upper limits are split by applying a lower boundary to the split restraint equal to *r*_*n*,*ij*_, the distance observed in the medoid structure. For splitting the lower limit, the upper boundary is set to *r*_*n*,*ij*_. By setting these lower and upper boundaries, the split restraints are prevented from violating the distances observed in the medoid structures. The standard deviations σ(*r*_*ij*_^noe^) for the eNOE are set to one since error estimates for eNOE restraints are already handled by upper and lower limits, which are split individually.

(v) Structure calculation for each *N* clusters individually, employing the split restraints *r*_*n*,*ij*_^noe^ where *n* = 1, ···, *N*. The ff14SB^55^ force field and the GB/SA implicit solvent model, implemented in AmberTools22,^54^ are applied to accurately describe the molecular forces.

### Structures of High Precision are Obtained for CsA with Hybrid (e)NOEs

The solution structure of CsA in CDCl_3_/*n*-hexadecane-*d*_34_ has been calculated by restrained simulated annealing torsion angle dynamics using CYANA. During restrained simulated annealing, lower and upper limit distance restraints have been applied from eNOE analysis, upper limit restraints (UPL) from semiquantitative NOE analysis, and combined semiquantitative NOE and eNOE restraints in the hybrid approach.

The hybrid method results in the highest precision, *i.e.*, lowest rmsd, structures (see Table 2). H-bond restraints between Aba 2 H and Val 5 O, Aba 2 O and Val 5 H, Dal 8 H and Mle 6 O have been applied. These hydrogen bonds were inferred from the temperature dependence of amide proton chemical shifts (Table S1). The chemical shifts of solvent exposed amide protons show a more pronounced temperature dependence than H-bonded amide protons.^75^ The threshold to discriminate between solvent exposed and internally H-bonded amide protons is generally considered to be −4.6 ppb/K. All CsA amide protons, with the exception of Ala 7 H, show a more positive shift than −4.6 ppb/K. For Ala 7 H, Δδ(^1^H)/Δ*T* = −7.36 ± 0.81 ppb/K, which is indicative of a solvent exposed amide proton. However, Ala H 7 is involved in a three centered H-bond^8^ as implied by X-ray crystallography and NMR structures. We speculate that the pronounced Δδ(^1^H)/Δ*T* for Ala H 7 is caused by sampling of additional conformations, an effect which has been described for proteins.^76^

**Table 2:** Number and Precision of Distance Restraints Used for Restrained Simulated Annealing of CsA Significantly Impacts the Precision of the Resulting Structures in CDCl_3_/*n*-hexadecane-*d*_34_^a^

	NOE	hybrid (e)NOE
total number of restraints	108	163
exact restraints	0	108
intraresidue |*i* – *j*| = 0	40	82
sequential |*i* – *j*| = 1	34	42
medium range |*i* – *j*| < 5	24	29
long-range |*i* – *j*| ≥ 5	10	10
rmsd/Å backbone	0.95	0.10
rmsd/Å	1.87	0.37

a The precision of the structures is given by the average rmsd from the mean structure for the bundle of 20 structures. 108 semi-quantitative constraints were obtained using the combined NOE assignment and structure calculation protocol, which is implemented in CYANA.^52^ 108 eNOE restraints were manually assigned.

To investigate the enhanced precision achieved through the utilization of eNOEs, restrained simulated annealing was performed with 56 semiquantitative NOE restraints and 56 eNOE restraints, both corresponding to the same proton-pairs. The resulting rmsd values are 1.25 (1.8) Å from eNOEs and 1.37 (2.3) Å from semiquantitative NOEs. The average rmsd from the mean structure of the 20 structures is given for the backbone and all atoms (in brackets). To show that the structures of CsA faithfully reproduce the hybrid (e)NOEs, the average restraints were back calculated from the bundle of structures and plotted against the experimental restraints (Figure S2).

High-precision structures of CsA in CDCl_3_ at 252.2 K have been determined through restrained molecular dynamics simulation by Kessler *et al.*(8) Although a direct comparison of their precision with ours is challenging due to differences in structure calculation methods (restrained molecular dynamics vs restrained simulated annealing) and differences in the applied force fields, we present a discussion in the following. Kessler *et al.* reported the rmsd values for both backbone and side chain dihedral angles. Consequently, we provide the corresponding rmsd values for CsA in Table S6B. The rmsd values for Ω and Φ are comparable, while for Ψ, the hybrid (e)NOE approach results in a slightly smaller rmsd of 2.40° compared to 3.36°. Additionally, the rmsds for the side chain torsion angles χ1 for residues Bmt 1, Mle 4, Val 5, Mle 6, Mle 9, Mle 10, Val11, and χ2 to χ4 of Bmt 1 are significantly lower for the hybrid (e)NOE structures. We did not attempt to compare the remaining dihedral angles due to the presence of different side chain populations, which were also reported by Kessler *et al.*(8)

In a more recent study, CsA structures in CDCl_3_ were refined by using residual-dipolar-coupling restraints in conjunction with NOE restraints.^77^ The reported all-heavy-atom rmsd is 0.12 Å for 10 of 20 calculated structures, while the corresponding rmsd for the hybrid (e)NOE structures is 0.25 Å. We note that the authors in Klages et al.^77^ mentioned that their structure is over-restrained.

**Figure 4:**
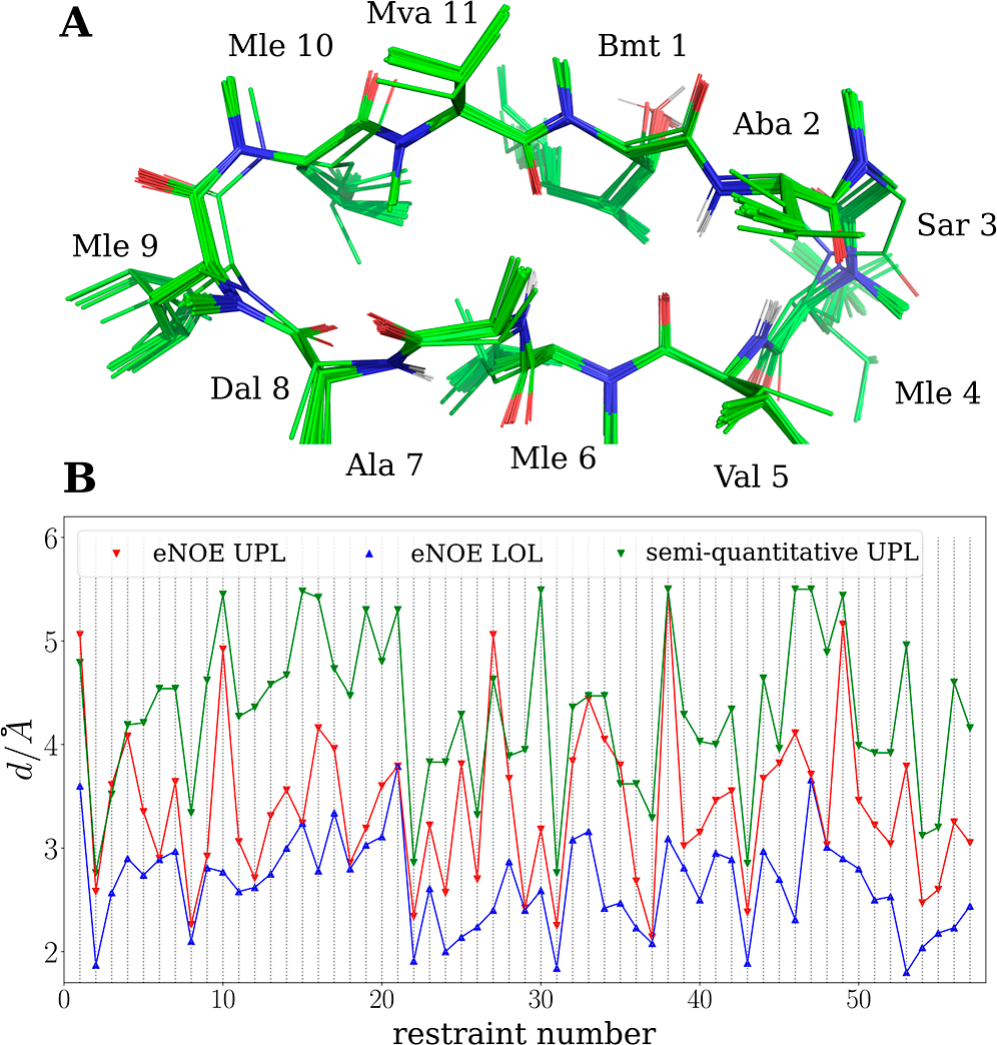
(A) The structure of CsA was determined using restrained simulated annealing, incorporating 163 hybrid (e)NOE distance restraints obtained from combining eNOE and semiquantitative NOE analysis. The resulting bundle comprises 20 structures that are superimposed on the mean structure. (B) Distance restraints from semiquantitative and eNOE analysis are shown. UPLs from semiquantitative NOEs are less restrictive than UPLs from eNOEs.

The overall features of the structures in Figure 4 are described previously for CsA:^8^ the peptide bond between residues 9 and 10 is in the cis state; and the molecule adopts an extended β sheet like structure. Three intramolecular H-bonds are implied by close contacts, *d*(H_donor_, O_accept_) < 2.1 Å, for Dal 8 H–Mle 6 O, Ala 7 H–Mva 11 O and Aba 2 H–Val 5 O. Notably, the side chain orientation for Bmt in the CsA structures presented here closely resembles the orientation observed by Kessler *et al.*, which was calculated through restrained molecular dynamics simulation. The Bmt side chain is folded over the backbone, similar to the conformation observed in the X-ray structure.^8^

In summary, using the hybrid (e)NOE protocol, a highly defined set of structures could be obtained with an rmsd of 0.10 Å for the backbone and 0.37 Å for all heavy atoms. The improvement in precision is most pronounced for the side chain atoms.

### OmphA Adopts Multiple Conformations with Distinct Exchange Kinetics

The protocols described in the previous sections were applied to study the conformations of OmphA in polar and apolar solvents.

### Apolar Environment: Cis–Trans Peptide Rotamers are in Slow Exchange

**Figure 5:**
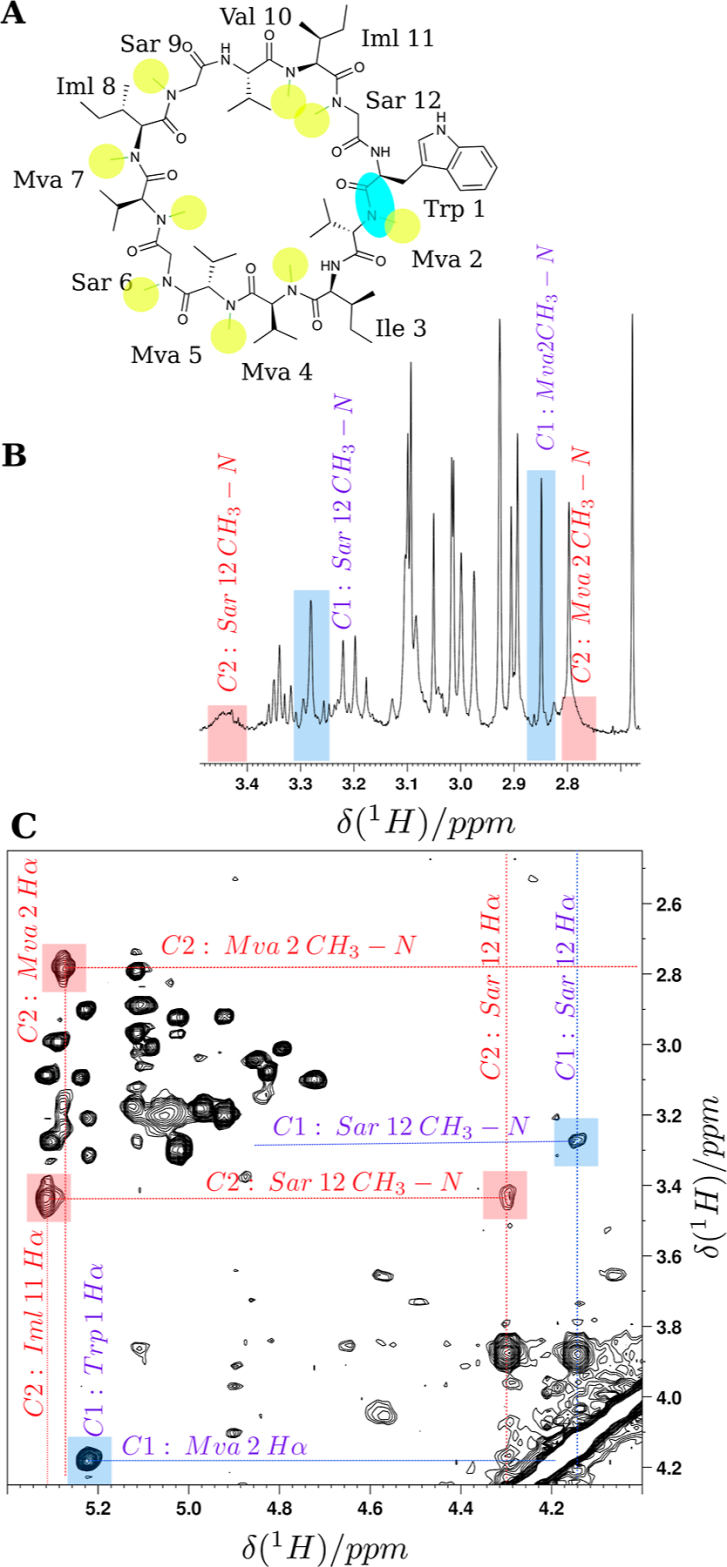
(A) The chemical structure of OmphA is shown with the peptide bond between Trp 1 and Mva 2 highlighted in cyan, and the *N*-methyl groups are highlighted in yellow. (B) The 1D ^1^H spectrum shows the spectral region for the CH_3_–N resonances of OmphA in CDCl_3_/*n*-hexadecane-*d*_34_. Signals for Sar 12 and Mva 2 are strongly broadened for C2 (indicated by the red boxes). (C) Two sets of signals are observable in the NOESY spectrum of OmphA in CDCl_3_/*n*-hexadecane-*d*_34_, one set for C1 (blue) and one set for C2 (red). Exchange signals between C1 and C2 are not observable in the NOESY spectrum (τ_mix_ = 400 ms). The exchange rate is estimated to *k*_ex_ < 10 s^–1^. The strong NOE between Trp 1 H^α^ and Mva 2 H^α^ is characteristic of a *cis* peptide bond. The strong NOE between Mva 2 H^α^ and Mva 2 CH_3_–N is indicative of the *trans* rotamer.

In the CDCl_3_/*n*-hexadecane-*d*_34_ solvent mixture, two distinct conformations for OmphA are observed, namely, C1 and C2, which are in slow exchange with approximately equal populations. In C1, the peptide bond between Trp 1 and Mva 2 adopts the *cis* rotameric state, indicated by the strong NOE cross-peak between Trp 1 H^α^ and Mva 2 H^α^. For C2, the strong NOE cross-peak between Mva 2 H^α^ and Mva 2 CH_3_–N is characteristic of the *trans* rotamer (Figure 5). For both C1 and C2, residues 4 to 11 demonstrate more pronounced line broadening compared to residues 1, 2, 3, and 12. This observation suggests that the residues near Trp 1 are less affected by conformational dynamics compared with the other parts of the macrocycle.

The temperature coefficient of the amide proton chemical shift, Δδ(^1^H)/Δ*T*, for Ile 3 H and Trp 1 H are −0.50 ± 0.11 ppb/K and −2.43 ± 0.03 ppb/K, respectively (Table S2). H-bond restraints have, therefore, been applied for Ile 3 H–Sar 12 O and Trp 1 H–Iml 11 O during restrained simulated annealing. In the resulting structures, distances between Ile 3 H and Sar 12 O are below 2.1 Å in 18 out of 20 structures. The observed H-bond between residues *i* and *i* + 3 confers stability to the type VIa β-turn. Distances below 2.1 Å between Trp 1 H and Iml 11 O are observed in 14 of 20 structures from restrained simulated annealing (Table S7). The H-bond between Trp 1 H and Iml 11 O, a characteristic for γ-turn, is therefore less stable than the H-bond between Ile 3 H and Sar 12 O. Overall, The backbone atoms of C1 form a β-sheet-like structure with the polar residues buried in the interior of the molecule.

Restraints for the indole side chain atoms of Trp 1 were not applied during restrained simulated annealing since the corresponding cross-peaks are not observable or overlapped with the residual CHCl_3_ signal. The orientation of the Trp 1 indole ring is, therefore, not defined by experimental restraints. To nevertheless gain insights into the orientation of the indole side chain of OmphA, we investigated the W1A variant. The ^1^H spectrum of W1A in CDCl_3_/*n*-hexadecane-*d*_34_ shows the presence of a conformation resembling C1 (Figure S3). A CH_3_–N signal at 3.28 ppm, shifted downfield, closely matches the chemical shift of Sar 12 in C1. By assuming that this signal originates from Sar 12 in OmphA W1A, we deduce that the corresponding protons in OmphA are minimally affected by ring current effects from the indole side chain. Therefore, the Trp 1 side chain is distantly located from CH_3_–N, a conclusion that aligns with the C1 structures obtained from hybrid (e)NOEs: the indole ring in the representative structure for C1 is positioned on top of Trp 1 H and distant to Sar 12 CH_3_–N [*d*(C^γ^, CH_3_) = 6.3 Å] as well as Mva 2 CH_3_–N [*d*(C^γ^, CH_3_) = 5.8 Å].

**Figure 6:**
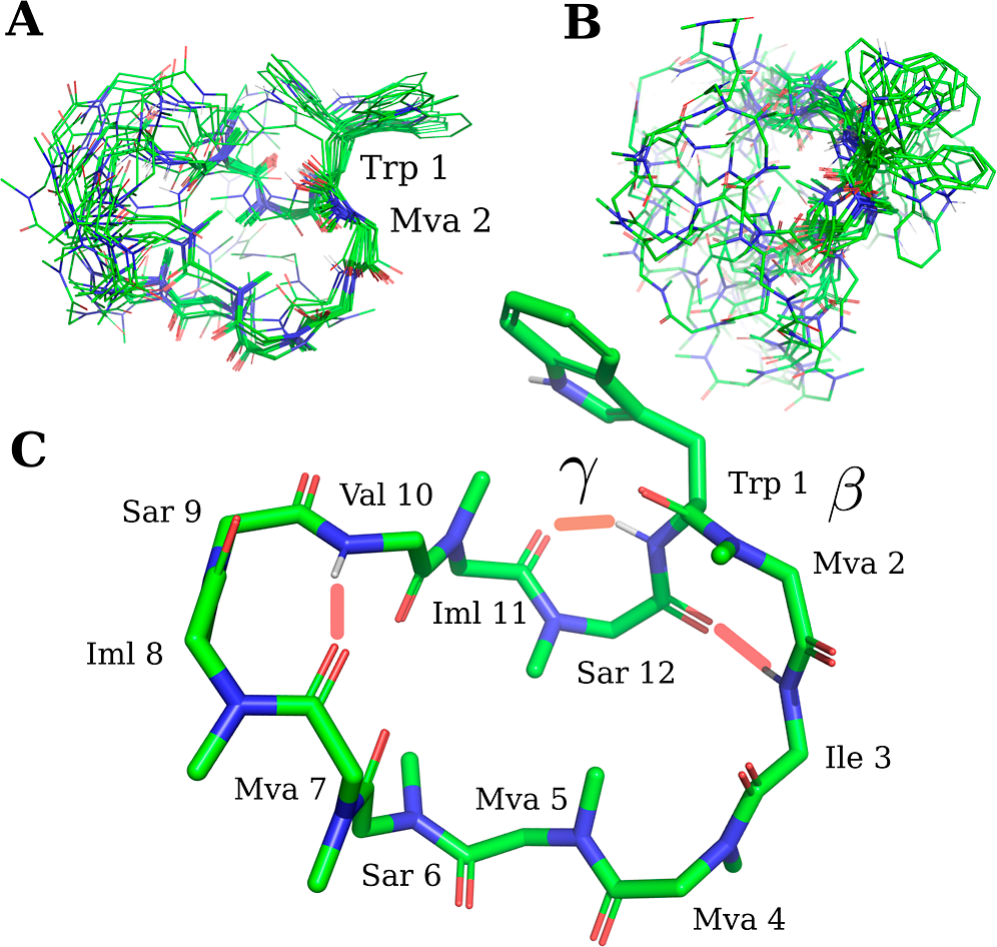
Backbone and the Trp 1 side chain atoms of OmphA in CDCl_3_/*n*-hexadecane-*d*_34_ are shown for conformations C1 (A) and C2 (B). The 20 top-ranked structures, ranked according to the restraints violation penalty, are superimposed to the backbone atoms of residues 1, 2, 3, 11, and 12 of the mean structures. Residues close to Trp 1 show a lower rmsd compared to residues 4 to 10. (C) The top-ranked structure for C1 is shown as a stick model. The orientation of the Trp 1 indole ring is undefined because of missing NOE distance restraints. However, the Trp 1 H resonance is upfield shifted (6.35 ppm) due to aromatic ring current effects, indicating that Trp 1 H is predominantly oriented toward the center of the indole side chain. Potential H-bonds (red bars) in C1 are between Trp 1 H and Iml 11 O, Ile 3 H and Sar 12 O, Val 10 H and Mva 7 O.

The structures in the bundle of C2 in CDCl_3_/*n*-hexadecane-*d*_34_ show poor precision, with an rmsd of 1.21 Å to the mean structure for the backbone atoms of residues 1, 2, 3, 11, and 12. The low precision of structures from restrained simulated annealing results from the low density of restraints (Table 3), which in turn is a result of line broadening. Line broadening indicates conformational heterogeneity in solution. Especially the resonances for Sar 12 and Mva 2 CH_3_–N show a pronounced line broadening at 278 K, which is reduced at 288 K as a result of faster conformational averaging at higher temperatures. None of the 20 structures in the C2 bundle show distances between H-bond donors and acceptors below 2.1 Å. Figure 6 shows the structures for C1 and C2, which have been calculated by restrained simulated annealing and subsequent refinement using hybrid (e)NOEs restraints (Table 3).

**Table 3:** Distance and Torsion Angle Restraints Utilized for the Structure Calculation of OmphA are Shown Together with the rmsd^a^

	CDCl_3_/*n*-hexadecane-*d*_34_		CD_3_OH/H_2_O
number of distance restraints	C1	C2	
total	96	27	134
exact restraints	46	26	73
intraresidue |*i* – *j*| = 0	47	14	54
sequential |*i* – *j*| = 1	38	10	61
medium range |*i* – *j*| < 5	2	1	11
long-range |*i* – *j*| ≥ 5	9	2	8
number of torsion angle restraints	5	2	3
rmsd/Å	0.49	1.21	0.97^#^, 0.99^+^

a The averaged pairwise rmsd is calculated for the backbone atoms (C, N, Cα) of residues 1, 2, 3, 11, and 12 to the mean coordinates. # “indole-out”, + “indole-in”.

Differences in structure and dynamics between C1 and C2 are most pronounced for residues 1, 2, 3, 11, and 12, as observed by the line shapes, the torsion angle ω_(Trp1–Mva2)_, and the differences in chemical shifts (Table S11). We propose that the formation of a hydrogen bond between Sar 12 O and Ile 3 H stabilizes the VIa β-turn in C1, thereby reducing the conformational dynamics of OmphA. The *cis* rotamer for ω_(Trp1–Mva2)_ promotes the formation of the Sar 12 O–Ile 3 H hydrogen bond. However, in the *trans* state, the increased distance between Sar 12 O and Ile 3 H destabilizes this hydrogen bond. Interestingly, exceptionally stable *i*, *i* + 3 H-bonds in *cis* type VI β-turns have been observed in peptide mimics.^78^

### Polar Environment: Trp 1 In- and Out-State in Fast Exchange

The backbone chain of OmphA resembles a β-sheet in CD_3_OH/H_2_O. The torsion angle ω_(Trp1–Mva2)_ = 0° (*cis*), as evidenced by the strong NOE cross-peak between Trp 1 H^α^ and Mva 2 H^α^, similar to conformation C1 observed in apolar solvents. However, unlike C1, the temperature dependence of the chemical shift for Ile 3 H, Δδ(^1^H)/Δ*T* = −7.10 ± 0.21 ppb/K, suggests that Ile 3 H is solvent-exposed and not involved in an intramolecular H-bond. Structures resulting from restrained simulated annealing, which show distances below 2.1 Å for Ile 3 H and Sar 12 O, are observed in 6 out of 20 structures. For Trp 1 H, Δδ(^1^H)/Δ*T* = −7.38 ± 0.37 ppb/K, and distances below 2.0 Å between Trp 1 H and Iml 11 O are not observed in the bundle of structures. This indicates that H-bond formation with solvent molecules is favored over the formation of intramolecular H-bonds for OmphA in CD_3_OH/H_2_O.

Resonances for residues 4 to 11 show pronounced line broadening, indicating intermediate conformational averaging on the NMR chemical shift time scale. Our results are largely consistent with the findings of Lopez,^79^ who reported a β-sheet-like NMR structure for OmphA in CD_3_OH at 240 K with the Trp 1 indole side chain oriented toward Trp 1 H. However, in our studies, by applying eNOE restraints, we could determine distinct conformational states for the Trp 1 indole side chain.

For the side chain indole of Trp 1, eNOE restraints that are not consistent with a single state are observed. We therefore treated these restraints as averaged following the procedure described in the previous section. The summarized procedure is as follows: an ensemble of 100 structures was generated using restrained simulated annealing in CYANA, incorporating all 134 (e)NOE hybrid restraints. Among these, seven restraints for the indole side chain of Trp 1 show violations exceeding 0.2 Å in more than 80% of the structures (Table S4) and were thereafter treated as averaged restraints. To ensure exhaustive sampling of Trp 1 side chain conformations using semi-restrained simulated annealing, the averaged restraints were excluded. The resulting ensemble of 100 structures was grouped into two clusters, and a set of restraints was generated for each cluster by splitting the averaged restraints (Table S5) according to eq 7. Finally, each cluster underwent independent structure refinement using the split restraints, employing the ff14SB^55^ force field and the GB/SA implicit solvent model.^54^

**Figure 7:**
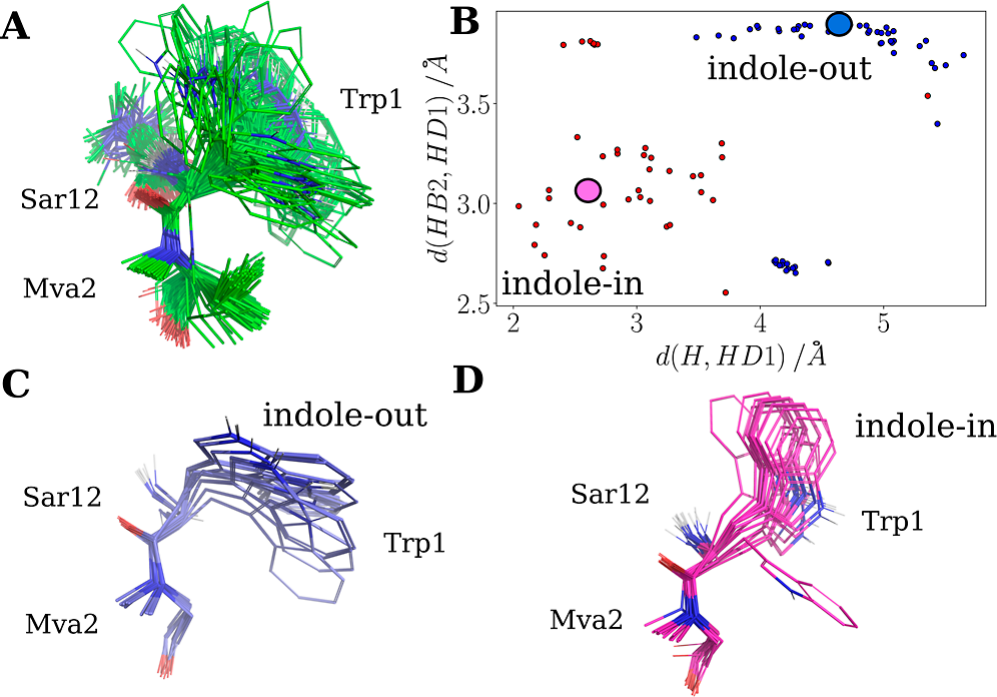
(A) The superposition of 100 structures from the bundle, calculated by semirestrained simulated annealing, is shown. The calculations were performed by omitting averaged restraints for OmphA in CD_3_OH/H_2_O. (B) The 100 structures have been grouped into two clusters using *k*-medoids clustering. The plot demonstrates that the cluster members, colored according to their cluster membership, occupy distinct regions in the distance space. These clusters correspond to two orientations for the Trp 1 indole side chain, termed the “indole-in” and “indole-out” state. Cluster centers, or medoids, are represented as magenta and blue disks for the “indole-in” and “indole-out” states, respectively. (C,D) Display the superposition of the 20 structures with the lowest restraint violation penalty for each corresponding state. In the “indole-in” state, the indole ring is oriented toward Sar 12, whereas in the “indole-out” state, it is oriented toward Mva 2. The average side chain torsion angles are as follows: χ1 = −140.8° and χ2 = −120.3° for the “indole-out” state, and χ1 = −69.5° and χ2 = −53.1° for the “indole-in” state. The backbone rmsd is 0.44 Å for both clusters. The structures are superimposed, and the rmsd is calculated based on the backbone atoms of residues 1, 2, and 12.

The resulting structures are shown in Figure 7. Two distinct states for the indole conformation are observed and labeled as “indole-in” and “indole-out”.

### Summary and Outlook

In this study, we demonstrated the enhanced precision of structure calculations for CsA and OmphA through the incorporation of distance restraints derived from NOE buildup rates and through the use of viscous solvent mixtures. In the case of CsA, utilizing semiquantitative upper limit distance restraints for restrained simulated annealing resulted in a backbone rmsd of 0.95 Å for the resulting structures. However, by employing our novel hybrid (e)NOE structure determination approach, we obtained high-precision structures with an rmsd of 0.10 Å for the backbone atoms. The improvement in the precision is especially reflected in all atom rmsd, which decreases from 1.87 to 0.37 Å.

OmphA poses a significant challenge due to its conformational flexibility and scarcity of NOEs. Nevertheless, we characterized distinct conformations of OmphA in both apolar and polar solvents, with interconversion rates ranging from fast to slow on the NMR time scale.

In apolar solvents, OmphA exhibits two slowly exchanging states, namely, C1 and C2. In C1, the torsion angle ω_(Trp1–Mva2)_ is *cis* (ω = 0°), while in C2, it is *trans* (ω = 180°). The *cis* state in C1 facilitates H-bond formation between the backbone atoms of Sar 12 and Ile 3, which contributes to the stabilization of a VIa β-turn. Additionally, C1 favors the formation of an γ-turn involving Iml 11 O as the H-bond acceptor and Trp 1 H as the donor. The intramolecular H-bonding in C1 restricts its conformational flexibility compared to C2.

In polar solvents, we characterized two states in fast exchange for Trp 1, which we refer to as the “indole-in” and “indole-out” states. The structures of these two states were determined by the usage of our new approach, the sample-cluster-refine method, for exchange-averaged eNOEs.

**Figure 8:**
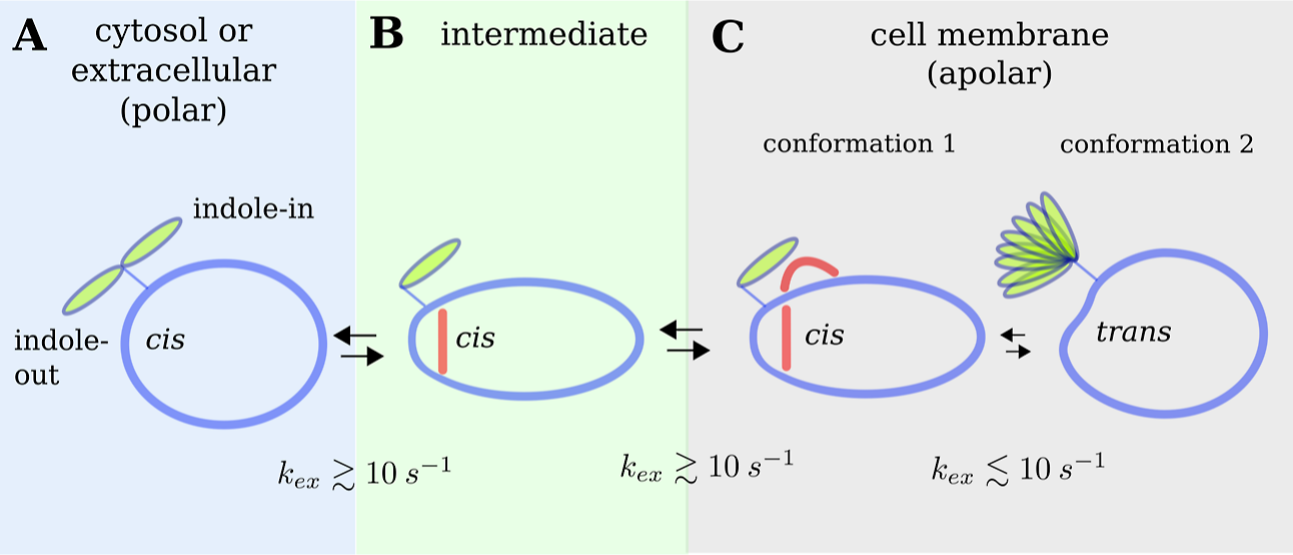
Schematic representation of the conformational states of the cation of OmphA. (A) An open state is observed in polar solvents with ω_(Trp1–Mva2)_ = 0° (*cis*). In polar solvents, H-bonds involving residues such as Ile 3 H, Sar 12 O, or Trp 1 H with solvent molecules are favored over intramolecular H-bonds, resulting in the presence of conformational substates that exchange rapidly. The indole side chain of Trp 1 exhibits fast exchange between an “indole-in” and an “indole-out” state. (B) An intermediate state is present in DMSO-*d*_6_/H_2_O. In this state, the torsion angle ω_(Trp1–Mva2)_ = 0° (*cis*), and a type VIa β-turn is stabilized by an *i* to *i* + 3 H-bond (red bar). However, a H-bond between Trp 1 H and Iml 11 O, characteristic of a γ-turn, is not observed (see Table S2 and Figure S6). We speculate that the exposed lipophilic indole side chain of Trp 1 serves as an anchor during membrane insertion. (C) Once OmphA crosses into the membrane, an additional H-bond (red semicircle) that stabilizes the γ-turn is formed, resulting in reduced polar contacts between C1 and the solvent. Transitions between the polar, intermediate, and apolar states occur rapidly (*k*_ex_ > 10 s^–1^), as no *cis*–*trans* conversion is involved. The transition between C1 and C2 in the apolar environment occurs slowly on the NMR time scale.

In the following, we present a model for membrane permeation of OmphA based on the structures obtained in polar, apolar, and mixed polarity solvents (Figure 8). In polar solvents, OmphA adopts an “open” conformation characterized by the absence of intramolecular H-bonds and a torsion angle ω_(Trp1–Mva2)_ = 0° (*cis*). The indole side chain of Trp 1 undergoes fast exchange between the “indole-in” and “indole-out” states. As a proxy to the structure in the intermediate polar–apolar environment, we have determined the structure of OmphA in DMSO-*d*_6_/H_2_O (Figure S6) using semiquantitative NOE restraints. In DMSO-*d*_6_/H_2_O, an H-bond is observed between Ile 3 H and Sar 12 O. However, no H-bond is indicated between Trp 1 H and Iml 11 O. These findings suggest that the transition to the hydrophobic conformation involves the formation of H-bonds, initiated by the formation of a type VIa β-turn in the intermediate state prior to entering the membrane. The indole side chain may serve as an anchor for membrane insertion.^80^ Within the lipophilic membrane, H-bond formation between Trp 1 H and Iml 11 O reduces the exposure of polar groups by the formation of a γ-turn. Furthermore, the indole side chain of Trp 1 is positioned toward Sar 12, resembling the “indole-in” state. A very similar type VI β and an adjacent γ-turn have been observed in cell permeable lariat peptides.^81^ The OmphA conformations in the polar solvents are in fast exchange with C1 in the hydrophobic environment since no high energy barrier crossing for a *cis* to a *trans* peptide bond isomerization is involved. It has been hypothesized that low-energy transitions between conformations in water and inside the cell membrane are required for passive membrane permeation.^3,82^ Within the apolar environment, the C1 ⇌ C2 transition involves crossing the high energy barrier between the *cis* and *trans* state of ω_(Trp1–Mva2)_. We speculate that C2 is not able to cross from the apolar to the polar environment directly but only after conversion to C1. The high energy barrier for the C1 ⇌ C2 transition may therefore increase the steady-state concentration of OmphA within the membrane.

Our findings indicate that the conformation of Trp 1 and its neighboring residues is distinct across various states of OmphA. In order to relate the conformational states found in this study to the binding-competent state, we propose a hypothesis for the target-bound conformation of OmphA. Since the target-bound conformation of OmphA is not known, we deduce the bound conformation from the OmphA variant OmphF. In OmphF, the oxidatively modified aromatic side chain of Trp 1 becomes rigidified due to the cyclization of the indole ring with the backbone.^83^

The nematotoxicity of OmphF is comparable to that of OmphA,^83^ suggesting that the rigidified tricyclic Trp 1 does not impede or even facilitate interaction with the target. Through model building and conformation analysis (Figure S5), we propose that the *cis* state is favored in OmphF due to the formation of H-bonds between the OH group of the tricycle and Iml 11 O, as well as between Ile 3 H and Sar 12 O. A similar H-bond network is observed in C1, where Trp 1 H serves as the H-bond donor for Iml 11 O. Our model suggests that the conformation of Trp 1 of OmphA C1 closely resembles that of OmphF. In conclusion, we hypothesize that an OmphA conformation resembling C1 in nonpolar solvents is capable of binding to the target.

We envisage that structures determined by utilizing the hybrid (e)NOE method will play an important role in guiding computational design methods in peptide macrocyclic drug discovery. Extensive conformational sampling and predicting populations for macrocyclic peptides is challenging. Conventional molecular dynamics approaches are burdened with high computational expenses and may result in incomplete sampling. The results often depend on the starting structure, which ideally should closely resemble the biologically relevant conformation but is often unknown.^84^ By providing starting structures from hybrid (e)NOEs, conformations close to the biologically relevant state are sampled more exhaustively.

To facilitate the docking of macrocyclic peptides with their respective targets, allowing flexibility in both the receptor and ligand is required.^85^ Docking of a ligand that closely resembles the bound state enhances the reliability. An NMR structure obtained from hybrid (e)NOEs in DMSO-*d*_6_/H_2_O may represent a valuable starting point for the structure of the bound state. Although the bulk properties of DMSO-*d*_6_/H_2_O resemble the binding epitope, the structures determined in DMSO-*d*_6_/H_2_O may not accurately represent the bound state. They lack spatial features specific to the epitope, and the macrocyclic peptide retains full flexibility. Nevertheless, we anticipate that such a structure will provide a valuable hypothesis for the bound state in many cases.

*N*-Hexadecane is commonly used in early drug discovery for predicting membrane permeability. A limitation of *n*-hexadecane is the absence of the polar headgroup, which could influence the localization of the drug within the membrane and its transition from the aqueous to the apolar phase. Alternatively, other common apolar solvents or lipid bilayers could be employed to investigate membrane-bound conformations.^86^

Temperature is another factor that can influence the conformations and dynamics of the macrocyclic drugs. Our studies have been performed at around 276 K, significantly below the physiological temperature. Consequently, we anticipate subtle structural differences, as it is well-established that temperature affects the populations in entropically driven equilibria.^87^ This temperature variation also offers the opportunity to explore the detailed energetics of conformations and transitions using hybrid (e)NOEs.

## Conclusions

We demonstrated that a detailed understanding of structure and dynamics can be obtained through the study of modified peptide macrocycles in both apolar and polar solvent mixtures. Our method exploits both eNOEs and semiquantitative NOEs. Furthermore, it combines extensive conformational sampling through restrained simulated annealing with accurate force fields and implicit solvent models for structure refinement. As a result, we developed a powerful NMR-based approach for investigating the structure of highly dynamic macrocyclic peptides, which are notoriously challenging to characterize. Based on the insights gained from our studies, we propose models for cell permeation and the binding-competent state of OmphA.

## Supporting Information Available

(ZIP)
Ruedisser2023_NMReNOE_supplementary.pdf: detailed procedures, NMR spectra, and tables with assignments and restraints (PDF)

## Notes

https://gitlab.ethz.ch/ruedsimo/hybrideNOE: scripts for data processing and structure calculation. pdb structures are deposited with the following pdb-code: CsA: 8Q7J; OmphA conformation 1: 8QAQ; OmphA “indole-Out”: 8QAS; OmphA “indole-In”: 8QBP. NMR data are available at the ETH research collection: DOI: 10.3929/ethz-b-000626950. The BMRB entry codes are the following: CsA: 34846; OmphA in apolar solvent: 34848; OmphA in polar solvent: 34849.
