# Supplementary material for: Conformations of Macrocyclic Peptides Sampled by Nuclear Magnetic Resonance: Models for Cell-Permeability
Source: J Am Chem Soc. 2023 Dec 8;145(50):27601–15. doi: 10.1021/jacs.3c09367 (PMC10739998; doi:10.1021/jacs.3c09367)
Supplement: Supplementary file 2 — ja3c09367_si_002.pdf [file ja3c09367_si_002.pdf]

Supporting information

Conformations of macrocyclic peptides sampled by NMR: models for cell-permeability

Simon H. Rüdisser [*] [†], Emmanuel Matabaro[†], Lukas Sonderegger[†], Peter Güntert[‡¶§], Markus Künzler[†], and Alvar D. Gossert [*] [†]

[†]Department of Biology, ETH Zürich, 8093 Zürich, Switzerland

[‡]Department of Chemistry and Applied Biosciences, ETH Zürich, 8093 Zürich, Switzerland

[¶]Institute of Biophysical Chemistry, Goethe University, 60438 Frankfurt am Main, Germany

[§]Department of Chemistry, Tokyo Metropolitan University, Hachioji, Tokyo 192-0397, Japan

Temperature dependence of the amide ^{1}H chemical shift for CsA

Table S1: The temperature dependence of the amide chemical shift for CsA is shown for the solvent mixture $CDCl_3$ / n-hexadecane-D_{34}.

	$\Delta\delta(^1H)\ \Delta T^{-1}$ / ppb K^{-1}
Aba 2 H	-3.75 + / - 0.36
Ala 7 H	-7.36 + / - 0.81
Val 5 H	-2.41 + / - 0.29
Dal 8 H	-1.90 + / - 0.40

Temperature dependence of the amide ^{1}H chemical shift for OmphA

Table S2: The temperature dependence of the amide chemical shift for OmphA is shown for different solvent mixtures.

	$CDCl_3$ / n-hexadecane-D_{34} $\Delta\delta(^1H)\ \Delta T^{-1}$ / ppb K^{-1}		CD_3OH / H_2O	DMSO-D_6 / H_2O
	C1	C2	$\Delta\delta(^1H)\ \Delta T^{-1}$ / ppb K^{-1}	$\Delta\delta(^1H)\ \Delta T^{-1}$ / ppb K^{-1}
Trp 1 HE1			-3.75 + / - 0.00	-5.12 + / - 0.18
Trp 1 H	-2.43 + / - 0.03		-7.38 + / - 0.37	-7.15 + / - 0.19
Ile 3 H	-0.50 + / - 0.11		-7.10 + / - 0.21	-1.74 + / - 0.46
Val 10 H			-8.85 + / - 0.11	-8.26 + / - 0.26

Investigating the influence of the T_1 relaxation delay (pre-scan delay) on (e)NOE restraints

In order to investigate whether the relaxation delay (pre-scan delay, d1 parameter) has an influence on the (e)NOE distance restraints, we have acquired two sets of NOESY spectra for CsA in $CDCl_3$ / n-hexadecane-D_{34} at 274 K. One set of experiments was acquired with a d1 delay of 1.5 s, the other set with a d1 delay of 10.0 s. Each set consists of 10 NOESY experiments acquired with the following NOE mixing times: 60, 100, 150, 200, 250, 300, 350, 400, 450, 500 ms. Note that the data acquired with d1 = 10.0 s were used to obtain the structures of CsA as described in the main article.

Figure S1: (A) UPLs and **(B)** LOLs, obtained from 2D NOESY spectra acquired with a d1 = 1.5 s and d1 = 10.0 are shown. **(C)** The difference, UPL – LOL, for d1 = 1.5 s is plotted versus the corresponding value for d1 = 10.0 s.

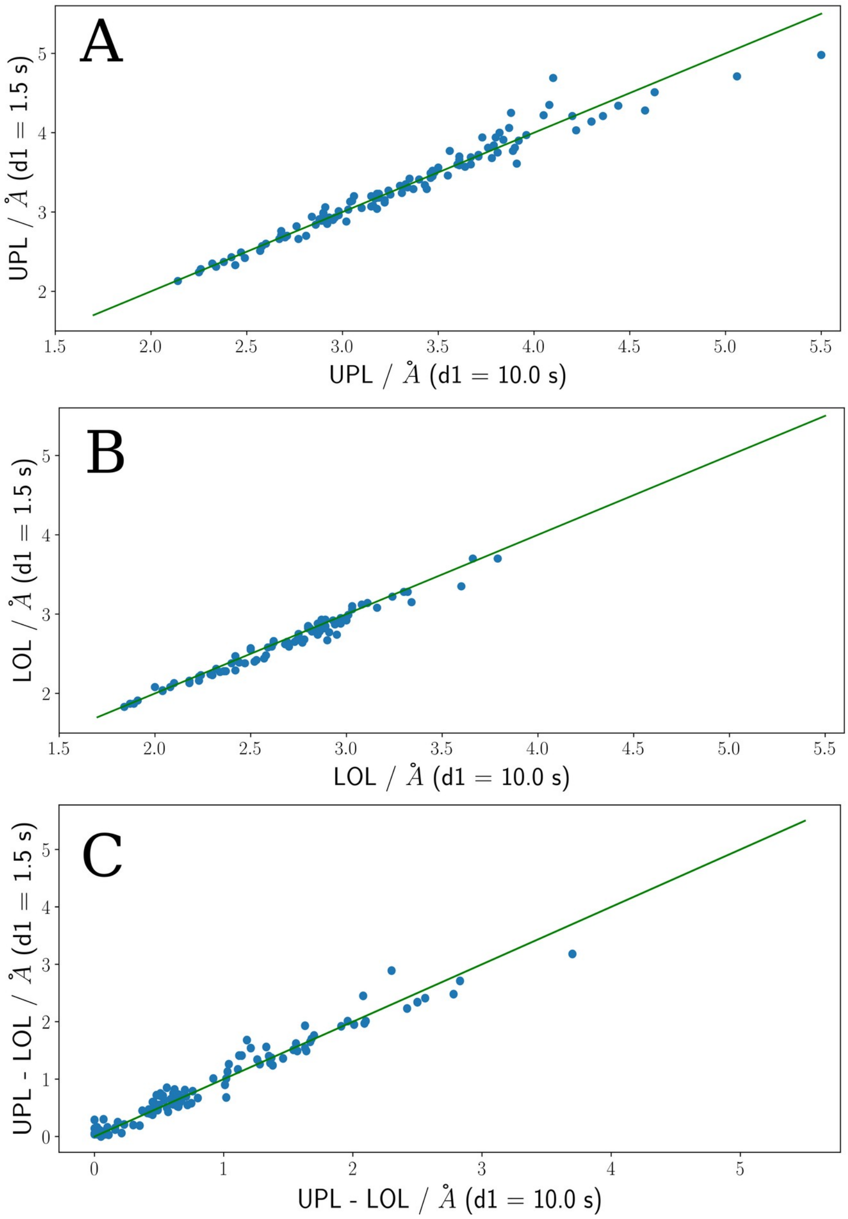

Figure S2: Upper- and lower limit (e)NOE distance restraints (UPL, LOL) for CsA are plotted versus the corresponding r^{-6} averaged distance in the bundle of 20 structure. The NOESY data for CsA were acquired in $CDCl_3$ / n-hexadecane-D_{34} at 274 K. The solid lines show the y-shifted diagonal with a shift of +0.2 Å and -0.2 Å for the blue and red line, respectively. The lower limit of 1.8 Å corresponds to the default value of semi-quantitative NOE restraints. Note that three LOLs are violated. These violations can be attributed to multiple states. However, we did not attempt to perform a multi-state analysis for CsA for these isolated restraints.

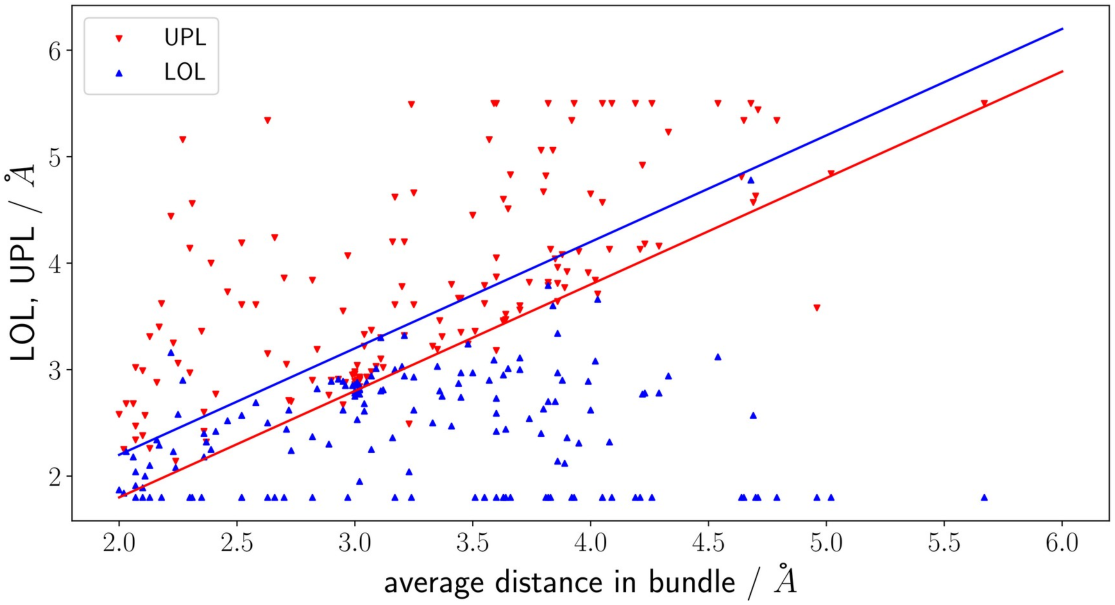

OmphA W1A variant

Figure S3: The ^{1}H NMR spectra of OmphA and the W1A variant of OmphA are shown for the sample in $CDCl_3$ / n-hexadecane-D_{34}. The W1A variant shows 11 signals for the CH_3-N protons with about equal intensity and at least one signal with strong line broadening. The assignments for OmphA are shown for the spectrum at 278 K. The downfield shifted Sar 12 CH_3-N signals for C1 and C2 are highlighted in yellow and blue, respectively.

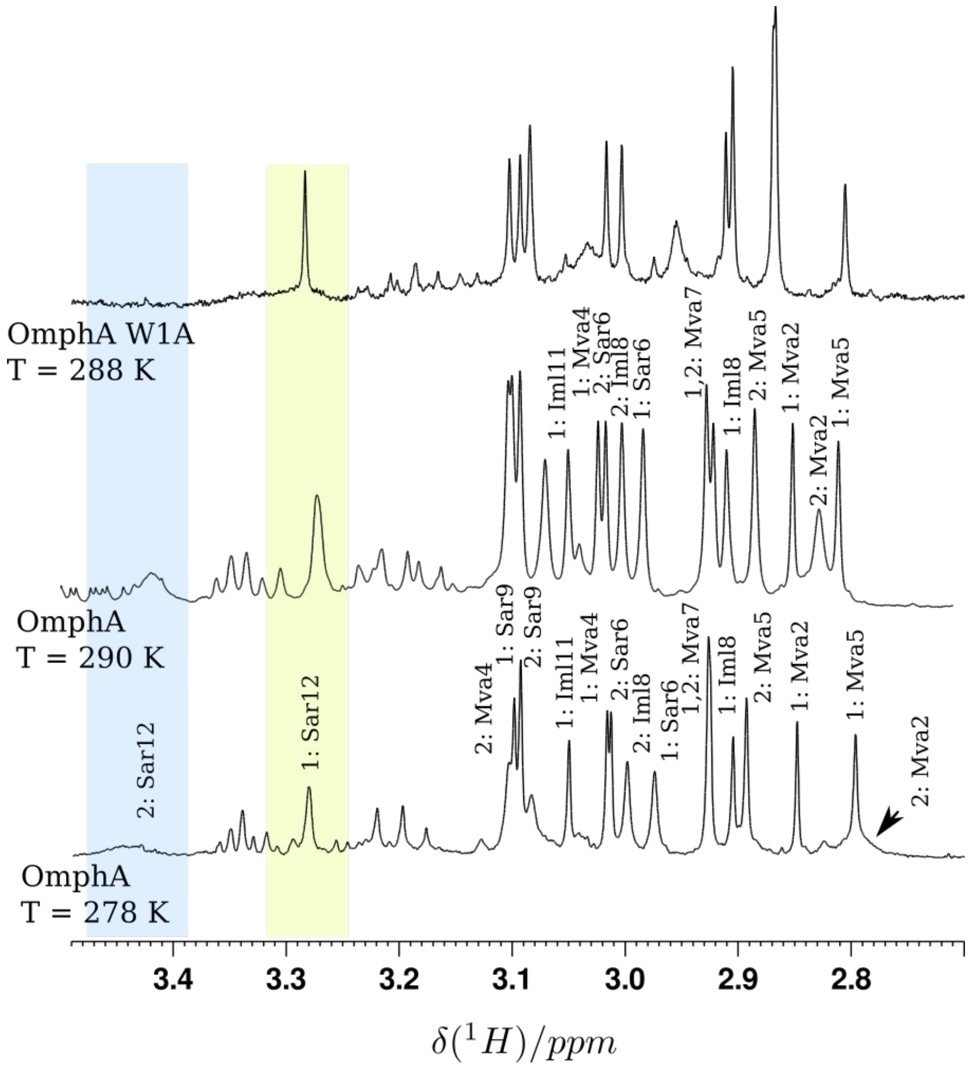

Figure S4: The ^{1}H NMR spectra of OmphA and the W1A variant of OmphA in CD_3OH / H_2O are shown. The spectral region of the N-methyl signals shows nine signals for both macrocycles. The N-methyl signal of Sar 12 is strongly broadened due to conformational exchange for OmphA.

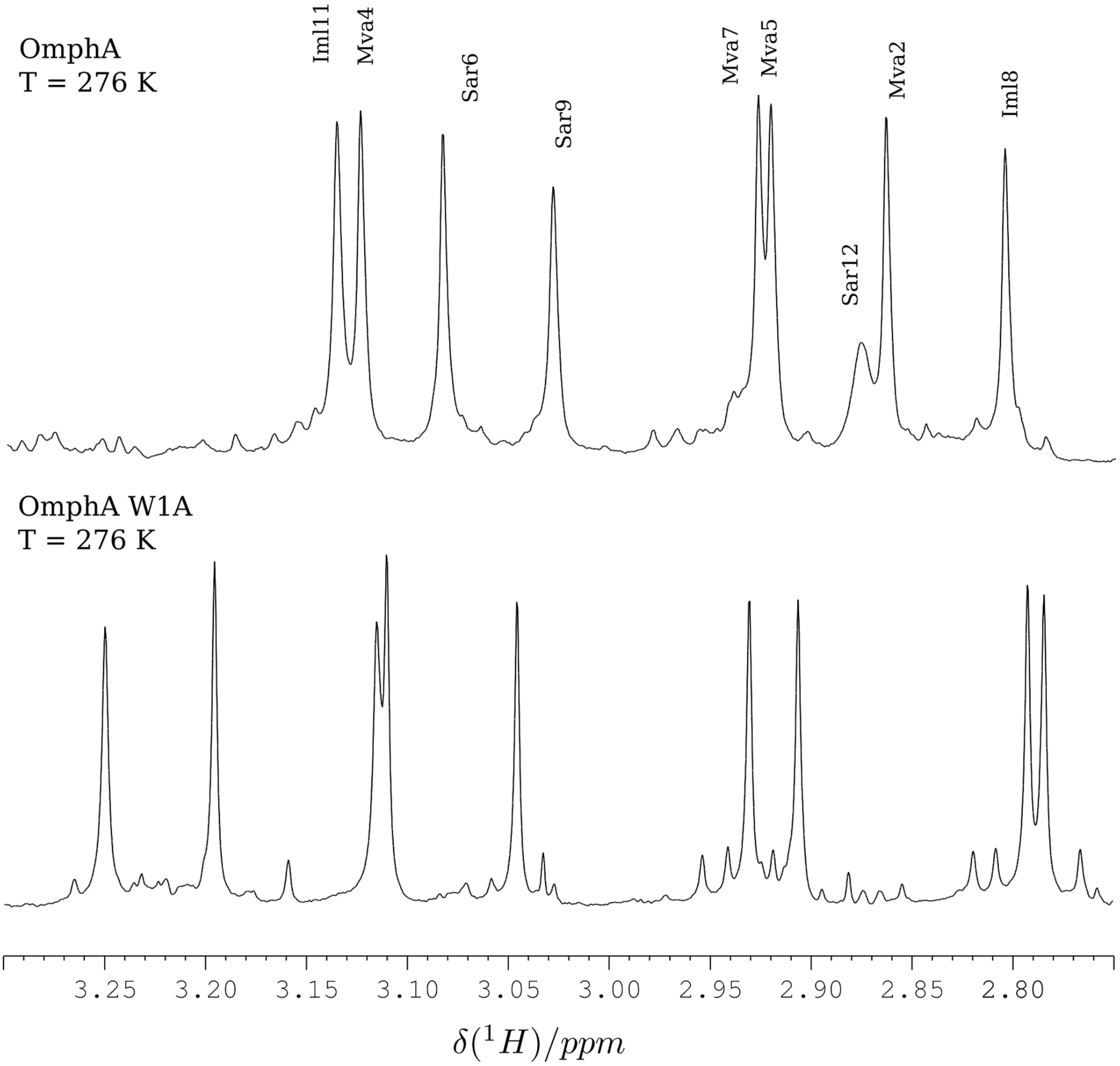

Structural model of Omphalotin F (OmphF)

Figure S5: The modeled structure of OmphF and the NMR structure of OmphA C1 in $CDCl_3$ / n-hexadecane-D_{34} are shown. The structure of OmpF has been modeled using C1 as a template and has been energy minimized (minimax in Witnotp[1]) using the TAFF[2] force field. A hydrogen bond between Trp 1 H and Iml 11 O, characteristic for a γ-turn, is indicated for OmphA. A potential hydrogen bond between Iml 11 O and the OH group of the tricyclic tryptophan derivative could stabilize a conformation which resembles a γ-turn.

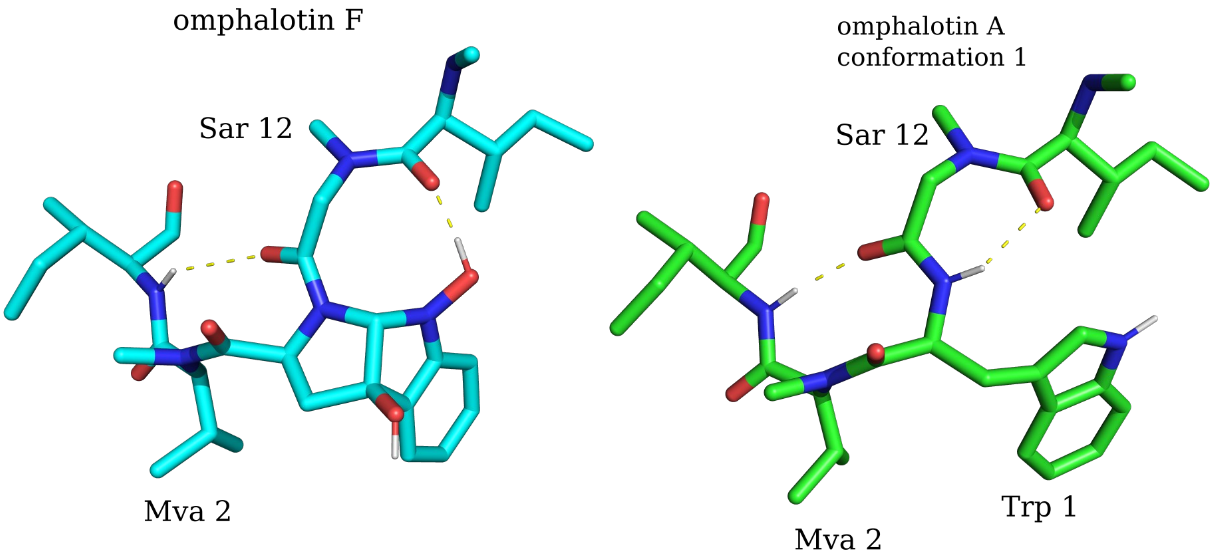

NMR structure of Omphalotin A in DMSO-D$_6$ / H$_2$O (90 %/ 10 % vol/vol) at 298 K

Figure S6: (A), (B): 2D NOESY spectrum of OmphA in DMSO-D$_6$ / H$_2$O. The amide proton region of the spectrum is shown in **(A)**. The intense cross peak between Trp 1 Hα and Mva 2 Hα is highlighted in **(B)**. **(C)** shows a representative NMR structure with the hydrogen bond between Ile 3 H and Sar 12 O indicated as red bar. **(D)**: 1D ^{1}H spectrum of the CH$_3$-N region. Assignments for the CH$_3$-N signals are from Sterner *et al.*[3]. Signals marked with a * are from impurities.

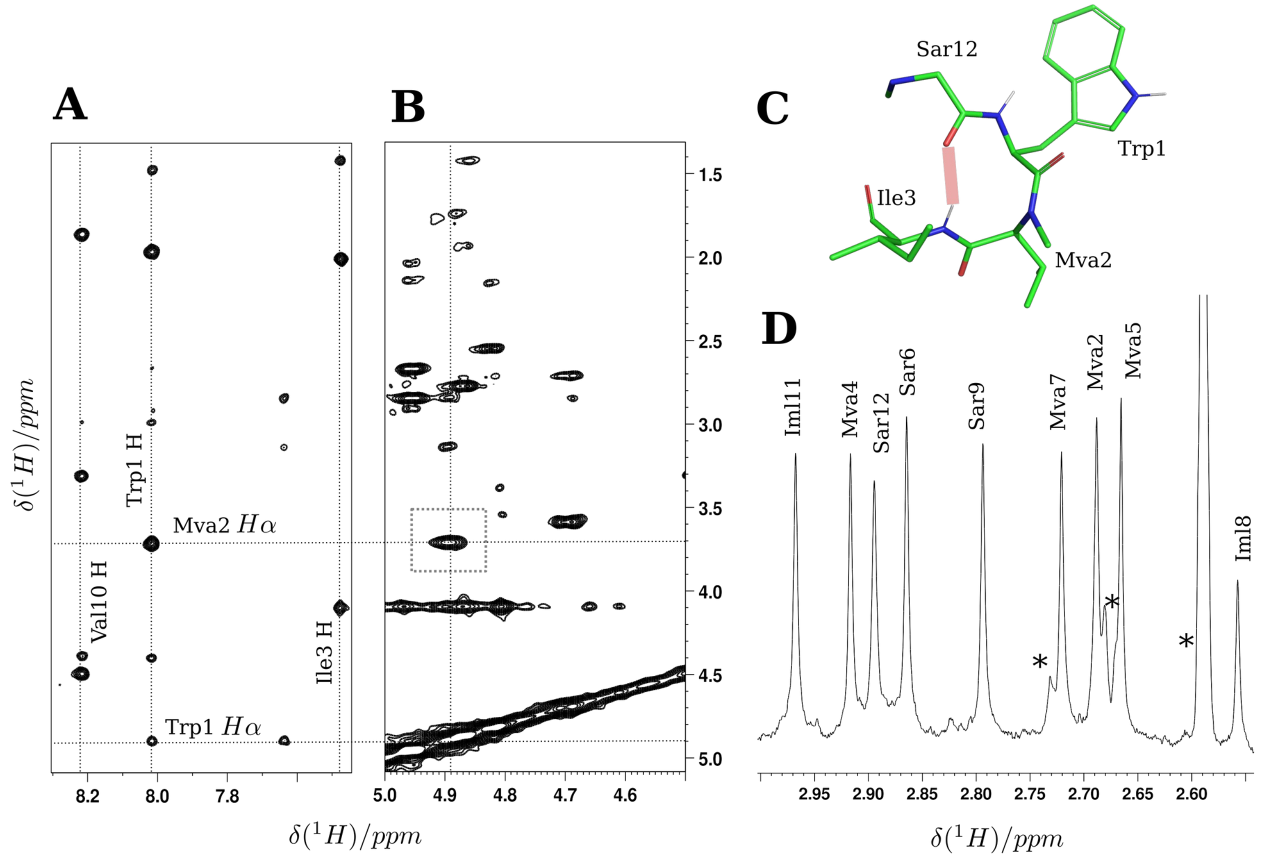

Table S3: Semi-quantitative NOE restraints which were applied during restrained simulated annealing for the structure calculation (noeassign macro in CYANA[4]) of OmphA in DMSO-D_6 / H_2O. NOE data were acquired at a mixing time of 90 ms to minimize spin diffusion. Restraints were applied for residues 12, 1, 2 and 3. H-bond restraints was inferred from the temperature dependence of the Ile 3 H chemical shift (Table S2).

**NOE distance restraints**		d / Å
1 TRP HA	3 ILE H	4.07
2 MVA HA	3 ILE H	2.94
1 TRP HA	2 MVA HA	2.98
3 ILE H	3 ILE HB	3.95
1 TRP H	1 TRP QB	3.23
1 TRP H	12 SAR QA	3.09
12 SAR QN	12 SAR QA	3.1

**H-bond restraints**		d / Å
3 ILE H	12 SAR O	2
3 ILE N	12 SAR O	3

**J coupling restraints**		J / Hz
1 TRP H	1 TRP HA	8
3 ILE H	3 ILE HA	8.5

Hybrid (e) NOEs: combining semi-quantitative and eNOE restraints

Fitting of the NOE buildup and decay data has been done using the eNOE (ENORA[5]) module in CYANA[4]. The output file from CYANA/ENORA (ovw-file) contains the restraints obtained from fitting each cross-peak individually. Corrections for spin-diffusion were applied within ENORA. The restraints were extracted from the ovw-file using self-written Python3[6] scripts and converted into upper- and lower-limit restraints (UPLs and LOLs). Corrections for methyl groups and uni-directional restraints were applied as described by Strotz *et al.*[5]

The multiplicative correction factors are (Strotz *et al*.[5]):
Methyl UPL: 0.833 * 1.085
Methyl LOL: 0.833 * 0.915
Unidirectional UPL: 1.2
Unidirectional LOL: 0.8

Restraints were merged using the merge method (outer merge) in pandas1.5.3[7]

Semi-quantitative NOE restraints were appended to the list of eNOE restraints to create hybrid (e)NOE restraints.

Ensemble averaged eNOE restraints for OmphA in CD_3OH / H_2O

Table S4: Restraint violations involving the indole protons of Trp 1 are observed in more than 80 out of 100 structures. Structure calculations by restrained simulated annealing were performed in CYANA. All hybrid (e)NOE restraints were applied.

H_i	H_j	type	Percentage of structures with violations	restraint eNOE	mean violation	max violation
Trp 1 H	Trp 1 HD1	upper	85	4.05	0.32	1.38
Trp 1 HB2	Trp 1 HD1	lower	86	3.03	0.22	0.47
Trp 1 HB2	Trp 1 HE3	upper	91	3.03	0.37	0.43
Trp 1 HD1	Mva 2 QN	upper	91	5.50	0.63	0.79
Trp 1 HE3	Mva 2 HA	lower	93	3.72	0.73	0.88
Trp 1 HE3	Mva 2 QG1	upper	93	3.49	0.39	0.48
Trp 1 HZ2	Mva 2 QG1	upper	93	5.44 (NOE)	0.41	1.04

Figure S7: NOESY cross-peak volumes at various mixing times are displayed together with the buildup curves for OmphA in CD_3OH / H_2O. The data are shown for the two cross-peaks in blue and green. For the restraint Trp 1 HE3 – Trp 1 HA, only one of the cross-peaks could be analyzed. The corresponding restraints are shown in Table S4.

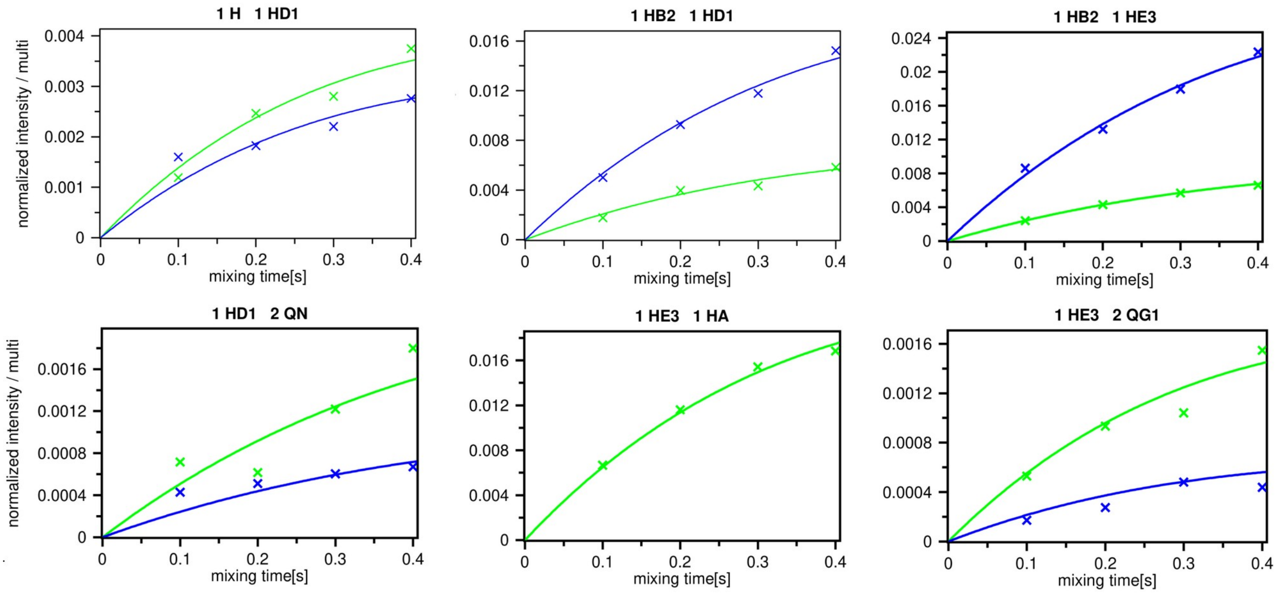

Clustering of structures, splitting of averaged eNOE restraints

100 structures from semi-restrained simulated annealing were clustered according to their proton-proton distances (Table S4). We have used the *k*-medoids method from the sklearn-learn[8] module for clustering. The number of cluster centers, *i.e.* medoids, has been optimized in order to achieve good cluster purity and separation[9] and to prevent over-fitting. The number of cluster centers was set to two in our application. Inspection of the distance sub-spaces (Figure S6) shows that the two clusters are well separated. To obtain upper limit restraints (UPLs) and lower limit restraints (LOLs) for both states, the averaged eNOEs were split by minimizing the target function described in equation (7). The minimize module from SciPy[10] was utilized.

Figure S8. Pairs of distances are plotted for 100 structures calculated by semi-restrained simulated annealing for OmphA in CD_3OH / H_2O. The data points are colored according to their cluster membership. The cluster medoids are shown as larger circles in cyan. Note that the cluster dissimilarity is computed in *n*-dimensional (*n* = number of proton-proton distances) space based on euclidean distance metric (Equation 6).

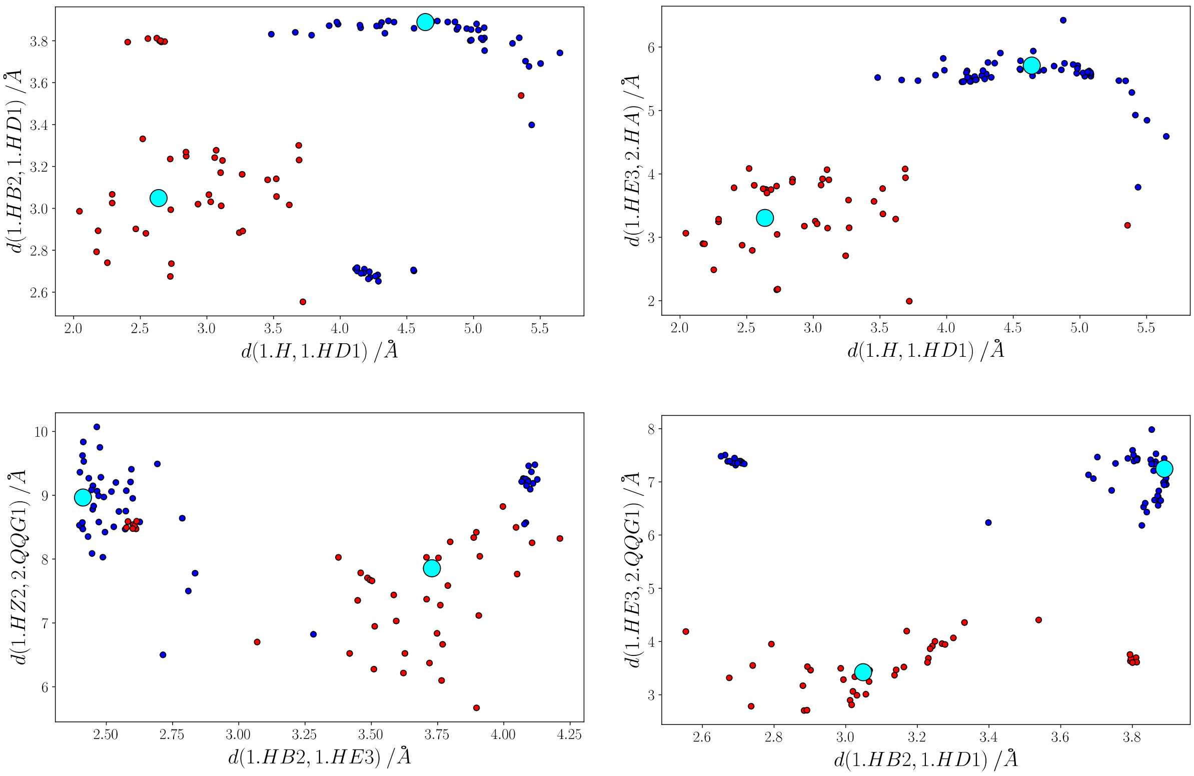

Table S5: The distance restraints obtained by splitting the averaged eNOEs are shown for the indole-in and indole-out state. For UPL > 5.50 Å, no restraints have been applied during restrained simulated annealing.

H_i			H_j			indole-out UPL / Å	indole-in UPL / Å	indole-out LOL / Å	indole-in LOL /Å
1	TRP	H	1	TRP	HD1	4.87	3.72	4.87	3.46
1	TRP	HB2	1	TRP	HD1	3.85	3.56	3.85	2.76
1	TRP	HB2	1	TRP	HE3	3.67	3.65	2.49	3.63
1	TRP	HD1	2	MVA	QN	5.01	6.95	1.8	1.8
1	TRP	HE3	2	MVA	HA	6.42	3.57	1.80	3.32
1	TRP	HE3	2	MVA	QG1	7.98	3.37	2.52	2.52
1	TRP	HZ2	2	MVA	QG1	5.50	6.52	1.8	1.8

Figure S9: Upper- and lower limit (e)NOE distance restraints (UPL, LOL) for OmphA are plotted versus the corresponding r^{-6} averaged distance in the bundle of 20 structure. The data are show for conformation 1 in apolar solvents **(A)**, the indole-in **(B)** and the indole-out **(C)** conformation. The solid lines show the y-shifted diagonal with a shift of +0.2 Å and -0.2 Å for the blue and red line, respectively. The lower limit of 1.8 Å corresponds to the default value of semi-quantitative NOE restraints. (A) shows no violation +/- 0.2 Å. For (B), the LOL restraint for Trp H – Trp Hδ1, and for (C), the LOL restraint for Trp Hβ2 – Trp Hε3, is violated. This indicates the presence of an additional minor state indicated by cluster members distant to the corresponding mediod (see Fig. S9).

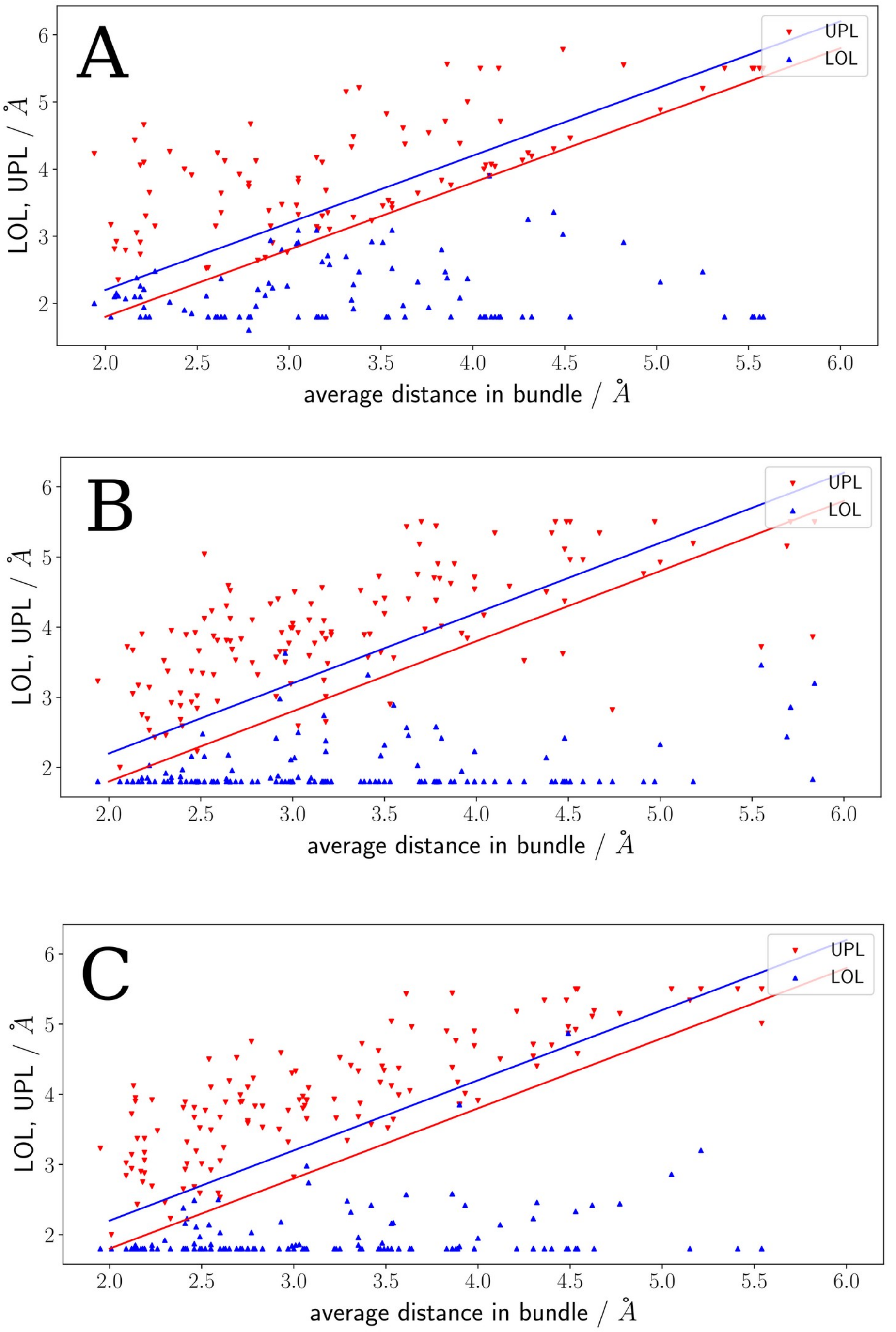

Table S6A: Torsion angles ω between residue *i* and *j*, H-bond distances and the distance restraint penalty for CsA in $CDCl_3$ / n-hexadecane-D_{34}. The data are shown for the 20 (out of 100) structures with the lowest restraint penalty. The structures have been calculated by restrained simulated annealing with the sander module from AmberTools[7].

nr	'(1 2)'	'(2 3)'	'(3 4)'	'(4 5)'	'(5 6)'	'(6 7)'	'(7 8)'	'(8 9)'	'(9 10)'	'(10 11)'	'(11 1)'	VAL5H ABA2O	ABA2H VAL5O	ALA7H MVA11O	DAL8H MLE6O	restraint penalty
1	-9	15	-17	-4	-11	2	3	-1	-174	-3	-2	2.12	1.87	1.79	2.13	15.7
2	-12	16	-16	-1	-7	-9	1	1	179	-5	-3	2.13	1.81	1.81	2.18	16.954
3	-9	16	-16	-3	-8	4	-2	-2	179	-3	-3	2.13	1.84	1.79	2.12	14.651
4	-10	15	-17	-5	-6	5	1	-3	-180	-3	-2	2.11	1.86	1.81	2.12	14.517
5	-10	15	-17	-2	-16	-8	2	-2	-179	-5	-2	2.13	1.81	1.81	2.2	17.094
6	-10	16	-17	-5	-6	5	0	-1	-180	-3	-3	2.12	1.86	1.79	2.1	14.89
7	-11	16	-17	-4	-8	1	-2	0	179	-3	-3	2.12	1.84	1.81	2.08	14.312
8	-10	16	-17	-4	-7	4	0	-2	179	-3	-2	2.12	1.84	1.81	2.11	13.907
9	-10	17	-17	-5	-7	4	-1	-1	178	-4	-3	2.12	1.85	1.81	2.1	14.229
10	-10	16	-17	-3	-9	0	-1	0	178	-3	-2	2.14	1.84	1.81	2.1	15.105
11	-10	14	-17	-3	-10	0	0	-1	179	-3	-2	2.1	1.86	1.8	2.1	15.696
12	-10	16	-16	-4	-7	3	-1	-2	179	-3	-3	2.13	1.86	1.82	2.12	14.641
13	-10	15	-17	-4	-10	3	2	-2	-180	-3	-2	2.12	1.86	1.8	2.11	16.217
14	-8	16	-17	-4	-8	3	-1	-2	179	-3	-1	2.13	1.85	1.82	2.11	13.666
15	-8	15	-19	0	-11	2	0	-3	179	-4	-3	2.09	1.85	1.81	2.1	16.427
16	-6	15	-19	-1	-10	1	-1	2	179	-4	-3	2.09	1.83	1.81	2.09	15.82
17	-9	15	-17	-4	-9	2	-1	-1	178	-4	-3	2.12	1.86	1.81	2.09	14.581
18	-6	15	-19	1	-11	1	1	-2	-180	-3	-3	2.1	1.85	1.81	2.09	16.801
19	-10	16	-17	-4	-7	3	3	2	177	-10	2	2.13	1.88	1.83	2.08	17.274
20	-5	5	15	-13	-13	4	2	-3	179	-3	-2	2.18	1.88	1.82	2.11	35.881

Table S6B: RMSD values for backbone and side chain torsion angles are shown for CsA in $CDCl_3$ / n-hexadecane-D_{34}. The data were calculated for the 20 (out of 100) structures with the lowest restraint penalty. The structures have been calculated by restrained simulated annealing with the sander module from AmberTools[7]. For comparison, the corresponding values from Kessler *et al.*[11] (marked with a #) are presented.

nr	residue	OMEGA	OMEGA#	PSI	PSI#	PHI	PHI#	CHI1	CHI1#
1	BMT	1.75	1.90	1.49	3.20	2.00	3.20	1.18	2.80
2	ABA	2.38	2.40	1.05	2.60	3.11	3.70	1.65	4.20
3	SAR	7.23	4.50	2.21	5.30	3.01	4.20		
4	MLE	2.80	3.10	3.74	4.30	3.18	5.00	4.55	13.30
5	VAL	2.51	2.00	2.56	2.70	3.85	5.70	7.93	2.50
6	MLE	3.79	1.00	3.87	3.00	6.04	2.20	1.80	2.30
7	ALA	1.48	1.80	2.33	3.40	0.99	3.90		
8	DAL	1.34	2.30	2.80	3.40	7.45	4.50		
9	MLE	1.86	3.80	2.19	2.30	3.29	2.20	0.82	2.60
10	MLE	1.50	3.10	1.31	3.70	3.92	2.40	1.87	9.50
11	MVA	1.19	1.90	2.87	3.10	4.38	2.90	1.40	2.40
**Average**		**2.53**	**2.53**	**2.40**	**3.36**	**3.75**	**3.63**	**2.65**	**4.95**

For BMT 1, the side chain torsion angles are the following:

CHI2	CHI2#	CHI3	CHI3#	CHI4	CHI4#	CHI5	CHI5#
0.92	2.70	0.92	3.80	1.82	17.10	30.80	2.30

Table S7: Torsion angles ω between residue *i* and *j*, H-bond distances and the distance penalty for OmphA C1 in $CDCl_3$ / n-hexadecane-D_{34}. The data are shown for the 20 (out of 100) structures with the lowest restraint penalty. The structures have been calculated by restrained simulated annealing with the sander module from AmberTools[12].

id	'(1 2)'	'(2 3)'	'(3 4)'	'(4 5)'	'(5 6)'	'(6 7)'	'(7 8)'	'(8 9)'	'(9 10)'	'(10 11)'	'(11 12)'	'(12 1)'	ILE3H SAR12O	TRP1H IML11O	VAL10H MVA7O	restraint penalty
44	-179	-9	-1	5	5	-4	4	-8	-11	8	-5	-13	2.01	2.1	6.7	1.429
31	178	-7	1	-4	2	-9	5	-6	-6	7	4	-3	2.02	2.07	6.74	2.652
70	178	7	11	-5	1	7	-179	-4	-10	4	-4	7	2.07	1.86	6.49	2.762
54	180	-8	3	-1	14	-10	178	-2	-4	-1	-2	0	2.01	2.04	5.18	3.094
19	176	2	4	-1	4	-10	7	-4	-3	8	4	-11	1.92	2.1	6.14	3.43
43	175	14	14	-6	4	1	4	-6	-3	0	0	1	1.98	1.95	5.65	3.482
71	180	-9	3	-2	5	1	3	14	-2	-6	0	-4	2	2.11	6.1	3.493
56	176	-5	2	-2	3	-10	7	-5	-4	6	4	-5	1.93	2.05	6.11	3.581
1	-179	-9	3	-2	13	-5	-177	0	-7	5	-2	-5	1.95	2.08	5.04	3.698
39	177	-2	-2	-13	2	5	-177	-13	1	9	-13	-2	1.91	2.15	7.64	3.936
92	179	-9	3	-1	15	-4	-7	-6	-2	-3	-1	0	1.98	2.02	2.46	4.031
88	173	18	2	4	8	-7	-177	0	0	-6	5	25	2.21	1.86	5.11	4.043
68	180	15	16	-5	3	-1	-2	-6	-3	0	1	2	2.07	1.95	3.88	4.231
21	-178	-8	3	-1	17	-6	-8	-12	7	0	3	-10	1.98	2.13	3.37	4.275
17	-179	-8	2	0	18	-8	177	-5	-3	0	2	-12	1.94	2.13	5.37	4.354
11	180	-4	2	-2	14	-6	-8	-2	-3	-1	-1	-5	2.05	2.03	2.3	4.472
46	176	13	12	-6	6	-2	-180	1	-12	5	-1	-1	1.93	2.01	6.57	4.523
14	-178	-7	2	0	16	-4	-176	-2	-5	0	3	-9	1.91	2.12	5.15	4.539
9	177	8	12	-5	2	-1	1	-6	2	1	-3	4	2.1	1.91	5.24	4.81
58	178	2	-8	7	20	-12	-8	-2	-1	5	-13	5	2.14	2.16	5.25	4.811

Table S8: Torsion angles ω between residue *i* and *j*, H-bond distances and the distance penalty for OmphA in CD_3OH / H_2O. The data are shown for the 20 (out of 100) structures with the lowest restraint penalty. The structures have been calculated by restrained simulated annealing with the sander module from AmberTools[7]. Restraints for the Trp 1 indole have not been applied (semi-restrained simulated annealing) to allow for extensive sampling of indole conformations.

id	'(1 2)'	'(2 3)'	'(3 4)'	'(4 5)'	'(5 6)'	'(6 7)'	'(7 8)'	'(8 9)'	'(9 10)'	'(10 11)'	'(11 12)'	'(12 1)'	ILE3H SAR12O	TRP1H IML11O	VAL10H MVA7O	restraint penalty
4	-179	-4	2	-6	8	-6	-11	1	12	7	-5	-1	3.65	4.95	2.38	5.282
36	-179	-4	-2	-9	-8	-1	8	6	-1	2	-1	10	3.39	2.98	4.89	5.864
71	179	5	6	-176	1	0	4	-7	2	-1	-174	3	3.62	6.13	5.88	5.98
17	171	13	-1	-1	177	-7	-1	5	-1	0	177	3	3.69	3.79	6.71	6.915
91	-178	0	-2	-9	-1	-1	3	8	-5	1	-1	-3	3.91	4.51	5.43	6.929
44	-177	-9	8	179	1	-4	1	172	-13	5	-173	7	3.62	3.93	5.92	7.157
60	173	-2	2	-3	4	-173	2	8	8	-6	1	1	3.46	1.98	5.83	7.191
25	174	-3	3	7	-3	-1	1	6	5	-5	178	-6	1.9	3.83	6.17	7.267
55	178	2	5	-180	-3	-2	13	175	-1	-4	174	2	3.56	3.85	8.18	8.131
12	178	0	-6	-179	4	5	2	-177	3	-6	-174	4	3.94	3.62	4.22	8.19
26	173	-1	1	2	2	3	-3	-4	1	-5	-172	-3	1.99	3.73	5.8	8.974
19	172	-1	4	-180	-5	-4	9	0	-2	-2	5	0	1.91	2.86	7.81	9.16
61	179	-6	3	-4	176	5	7	-9	-13	5	175	-3	3.9	4.61	5.24	9.651
34	-178	-14	3	0	6	0	12	178	-7	10	11	2	2.63	4.1	6.04	9.664
64	178	4	3	1	179	3	7	-5	-2	2	2	0	3.63	2.59	6.68	9.969
2	175	-8	5	-6	14	10	5	175	2	5	5	3	1.95	5.24	4.21	10.259
10	174	0	0	2	11	3	6	-175	5	6	7	3	3.35	2.09	5.21	10.35
41	173	5	-3	-5	176	178	-1	4	3	1	-179	1	3.68	3.82	6.13	10.43
40	175	-3	5	3	-175	180	3	-4	3	-2	9	0	1.94	5.24	5.36	10.431
28	174	-7	-1	-2	3	1	10	175	5	-8	16	5	1.94	5.22	8.09	10.776

CsA assignments

Table S9: Chemical shift assignments for CsA in $CDCl_3$ / n-hexadecane-D_{34} are shown. Q atoms denote chemically equivalent protons in methyl groups.

atom			δ / ppm
1	BMT	HA	5.53
1	BMT	HB	3.84
1	BMT	HD22	2.46
1	BMT	HD23	1.69
1	BMT	QD1	0.76
1	BMT	QH	1.66
1	BMT	QN	3.56
2	ABA	H	8.15
2	ABA	HA	5.08
2	ABA	HB2	1.75
2	ABA	HB3	1.71
2	ABA	QG	0.91
3	SAR	HA2	4.76
3	SAR	HA3	3.22
3	SAR	QN	3.44
4	MLE	HA	5.4
4	MLE	HB2	2.05
4	MLE	HB3	1.68
4	MLE	QN	3.15
4	MLE	QD1	0.91
5	VAL	H	7.55
5	VAL	HA	4.69
5	VAL	HB	2.49
5	VAL	QG1	1.11
5	VAL	QG2	0.92
6	MLE	HA	5.03
6	MLE	HB2	1.39
6	MLE	HB3	2.13
6	MLE	HG	1.85
6	MLE	QD2	0.86
6	MLE	QN	3.31
7	ALA	H	7.82
7	ALA	HA	4.58
7	ALA	QB	1.4
8	DAL	H	7.27
8	DAL	HA	4.87

atom			δ / ppm
8	DAL	QB	1.28
9	MLE	HA	5.74
9	MLE	HB2	2.21
9	MLE	HB3	1.24
9	MLE	HG	1.34
9	MLE	QN	3.16
10	MLE	HA	5.13
10	MLE	HB2	2.09
10	MLE	HB3	1.33
10	MLE	HG	1.51
10	MLE	QN	2.73
11	MVA	HA	5.14
11	MVA	HB	2.19
11	MVA	QG2	0.89
11	MVA	QN	2.75
11	MVA	QG1	1.05

CsA: distance restraints

Table S10: The distance restraints, UPLs (Upper Distance Limits) and LOLs (Lower Distance Limits), for CsA in $CDCl_3$ / n-hexadecane-D_{34} are shown. Restraints to pseudo-atoms Q have been applied for methyl and methylene groups.

H_i			H_j			LOL / Å	UPL / Å
1	BMT	QN	1	BMT	QD1	3.6	5.06
1	BMT	QN	1	BMT	HD23	2.44	3.47
1	BMT	QN	11	MVA	HA	1.87	2.58
1	BMT	QN	11	MVA	HB	2.76	3.35
1	BMT	QN	11	MVA	QG1	2.57	3.61
1	BMT	QN	11	MVA	QG2	2.9	4.08
1	BMT	HA	1	BMT	QN	2.36	4.2
1	BMT	HA	1	BMT	HB	2.32	2.32
1	BMT	HA	1	BMT	QD1	2.74	3.35
1	BMT	HA	1	BMT	HD22	2.89	2.9
1	BMT	HA	1	BMT	HD23	2.97	3.64
1	BMT	HA	2	ABA	H	2.1	2.26
1	BMT	HA	7	ALA	H	2.81	2.92
1	BMT	HA	11	MVA	QN	2.77	4.92
1	BMT	HB	1	BMT	QD1	2.58	3.06
1	BMT	HB	2	ABA	H	2.62	2.71
1	BMT	HB	3	SAR	QN	2.75	3.31
1	BMT	QD1	6	MLE	HA	3	3.56
1	BMT	HD22	1	BMT	QD1	2.62	4.66
1	BMT	HD22	6	MLE	HA	3.24	3.24
1	BMT	HD23	1	BMT	QD1	2.25	4
1	BMT	QN	11	MVA	QG1	1.8	4.19
1	BMT	QN	1	BMT	HA	1.8	4.58
1	BMT	HA	6	MLE	HA	1.8	3.86
1	BMT	HA	7	ALA	QB	1.8	5.5
1	BMT	HB	1	BMT	HD22	1.8	4.13
1	BMT	HB	1	BMT	HD23	1.8	4.13
1	BMT	HB	5	VAL	H	1.8	5.5
1	BMT	HD23	6	MLE	HA	1.8	5.48
1	BMT	HA	11	MVA	HB	1.8	4.81
1	BMT	QN	1	BMT	QD2	1.8	4.56
1	BMT	HA	1	BMT	QD2	1.8	3.84
1	BMT	HA	6	MLE	QB	1.8	5.34
1	BMT	HB	1	BMT	QD2	1.8	3.62

H_i			H_j			LOL / Å	UPL / Å
1	BMT	QD2	2	ABA	H	1.8	5.34
1	BMT	QD2	6	MLE	HA	1.8	4.62
2	ABA	H	2	ABA	HA	2.86	2.89
2	ABA	H	2	ABA	HB2	2.82	3.19
2	ABA	H	2	ABA	HG3	2.78	4.16
2	ABA	H	3	SAR	QN	3.34	3.96
2	ABA	H	5	VAL	H	2.8	2.86
2	ABA	H	5	VAL	HB	3.03	3.19
2	ABA	H	6	MLE	HA	3.11	3.6
2	ABA	H	7	ALA	H	3.79	3.79
2	ABA	HA	2	ABA	HB3	2.25	3.37
2	ABA	HA	2	ABA	HB2	2.77	2.84
2	ABA	HA	2	ABA	HG3	2.54	3.82
2	ABA	HA	3	SAR	QN	1.91	2.34
2	ABA	HB3	5	VAL	HB	1.8	5.5
2	ABA	HB2	5	VAL	HB	1.8	5.5
2	ABA	H	11	MVA	HB	1.8	5.5
3	SAR	QN	3	SAR	HA2	2.61	3.22
3	SAR	QN	3	SAR	HA3	2	2.57
3	SAR	HA2	4	MLE	QN	2.04	2.49
3	SAR	HA2	5	VAL	H	2.78	4.18
3	SAR	QN	3	SAR	QA	1.8	3.02
3	SAR	QA	4	MLE	QN	1.8	2.97
3	SAR	QA	5	VAL	H	1.8	5.34
4	MLE	QN	4	MLE	HA	3.03	3.78
4	MLE	QN	4	MLE	HB2	2.89	3.91
4	MLE	QN	4	MLE	HB3	2.36	3.92
4	MLE	QN	4	MLE	HG	2.14	3.81
4	MLE	QN	5	VAL	H	2.24	2.7
4	MLE	QN	5	VAL	QG2	2.4	5.06
4	MLE	HA	4	MLE	HB2	2.42	2.77
4	MLE	HA	4	MLE	HB3	2.68	3.33
4	MLE	HA	4	MLE	HG	2.7	4.04
4	MLE	HA	4	MLE	QD1	2.87	3.67
4	MLE	HA	5	VAL	H	2.8	3.1
4	MLE	HB3	5	VAL	H	4.78	5.5
4	MLE	QN	5	VAL	QG1	1.8	4.63
4	MLE	QN	5	VAL	HB	1.8	5.5
4	MLE	QN	4	MLE	QB	1.8	3.36
4	MLE	QN	5	VAL	QG	1.8	3.52
5	VAL	H	5	VAL	HA	2.85	2.88
5	VAL	H	5	VAL	HB	2.4	2.42

H_i			H_j			LOL / Å	UPL / Å
5	VAL	H	5	VAL	QG1	2.59	3.18
5	VAL	HA	5	VAL	HB	2.88	2.9
5	VAL	HA	5	VAL	QG1	2.37	2.9
5	VAL	HA	5	VAL	QG2	2.52	3.73
5	VAL	HA	6	MLE	QN	1.84	2.25
5	VAL	HB	4	MLE	QN	2.63	4.67
5	VAL	HB	5	VAL	QG1	2.18	2.68
5	VAL	HB	5	VAL	QG2	2.29	3.4
5	VAL	HB	6	MLE	QN	3.08	3.84
5	VAL	QG1	6	MLE	QN	3.16	4.44
5	VAL	QG2	6	MLE	QN	2.42	4.05
5	VAL	H	5	VAL	QG2	1.8	5.49
5	VAL	H	6	MLE	QN	1.8	5.5
5	VAL	H	5	VAL	QG	1.8	4.07
5	VAL	QQG	6	MLE	QN	1.8	3.62
6	MLE	QN	6	MLE	HA	2.93	3.61
6	MLE	QN	6	MLE	HB2	2.47	3.8
6	MLE	QN	6	MLE	HB3	2.23	2.68
6	MLE	HA	1	BMT	HD23	3.01	4.51
6	MLE	HA	6	MLE	HB2	2.69	3.61
6	MLE	HA	6	MLE	HB3	2.94	2.98
6	MLE	HA	6	MLE	HG	3.3	3.3
6	MLE	HA	6	MLE	QD2	2.32	4.13
6	MLE	HA	7	ALA	H	2.08	2.14
6	MLE	HA	11	MVA	QN	3.09	5.5
6	MLE	HB2	7	ALA	H	2.73	3.87
6	MLE	HB2	11	MVA	QN	2.94	4.2
6	MLE	HB3	6	MLE	HG	2.62	2.67
6	MLE	HB3	8	DAL	H	2.97	4.45
6	MLE	HG	6	MLE	QN	2.62	4.65
6	MLE	QN	6	MLE	HG	1.8	5.5
6	MLE	QN	7	ALA	H	1.8	5.5
6	MLE	HG	8	DAL	H	1.8	4.84
6	MLE	QB	8	DAL	H	1.8	5.34
7	ALA	H	6	MLE	QN	3.12	5.5
7	ALA	H	7	ALA	HA	2.91	2.91
7	ALA	H	8	DAL	H	2.81	3.02
7	ALA	H	11	MVA	QN	2.5	3.15
7	ALA	H	11	MVA	QG2	2.57	4.57
7	ALA	HA	8	DAL	H	2.85	2.95
7	ALA	H	7	ALA	QB	1.8	4.14
7	ALA	QB	11	MVA	QN	1.8	4.24

H_i			H_j			LOL / Å	UPL / Å
7	ALA	H	11	MVA	QG1	1.8	5.44
8	DAL	H	8	DAL	HA	2.78	2.96
8	DAL	H	8	DAL	HB3	2.95	3.46
8	DAL	H	11	MVA	QN	2.89	3.55
8	DAL	HA	8	DAL	HB3	1.95	2.93
8	DAL	HA	9	MLE	QN	1.89	2.38
8	DAL	HA	11	MVA	QN	2.97	3.67
8	DAL	HB3	9	MLE	QN	2.7	3.82
8	DAL	H	9	MLE	QN	1.8	4.89
9	MLE	QN	8	DAL	H	2.94	5.23
9	MLE	QN	9	MLE	HA	3	3.61
9	MLE	QN	9	MLE	HB2	2.3	2.76
9	MLE	HA	9	MLE	HB2	2.82	2.91
9	MLE	HA	9	MLE	HG	2.75	2.98
9	MLE	HA	9	MLE	QQD1	2.9	5.16
9	MLE	HA	9	MLE	QD2	2.31	4.11
9	MLE	HA	10	MLE	HB2	3.66	3.71
9	MLE	HA	10	MLE	HG	3.01	3.03
9	MLE	HA	10	MLE	QD1	2.9	5.16
9	MLE	HA	11	MVA	QN	2.8	3.46
9	MLE	HG	9	MLE	QN	2.12	3.77
9	MLE	HA	10	MLE	HB3	1.8	5.5
9	MLE	HA	9	MLE	QD1	1.8	5.5
9	MLE	HA	10	MLE	QN	1.8	4.82
9	MLE	QN	9	MLE	HG	1.8	4.45
9	MLE	HA	10	MLE	HA	1.8	2.99
9	MLE	QN	9	MLE	QB	1.8	3.31
9	MLE	HA	10	MLE	QB	1.8	4.83
10	MLE	QN	10	MLE	HB2	2.5	3.22
10	MLE	QN	10	MLE	HB3	2.53	3.04
10	MLE	QN	11	MVA	QG2	1.8	3.79
10	MLE	HA	10	MLE	HB2	2.85	2.9
10	MLE	HA	10	MLE	HB3	2.88	2.93
10	MLE	HA	10	MLE	HG	3.32	3.32
10	MLE	HA	11	MVA	QN	2.04	2.47
10	MLE	QN	11	MVA	QG1	1.8	4.96
10	MLE	QN	10	MLE	QB	1.8	3.36
10	MLE	QN	11	MVA	QG1	1.8	3.58
11	MVA	QN	11	MVA	HB	2.18	2.6
11	MVA	QN	11	MVA	QG1	2.84	4.46
11	MVA	QN	11	MVA	QG2	2.23	3.25
11	MVA	HA	11	MVA	QG1	2.71	3.52

H_i			H_j			LOL / Å	UPL / Å
11	MVA	HA	11	MVA	QG2	2.44	3.05
11	MVA	HB	11	MVA	QG1	2.48	3.34
11	MVA	HB	11	MVA	QG2	2.34	2.88
11	MVA	QN	11	MVA	QG1	1.8	4.6
11	MVA	HA	11	MVA	QG1	1.8	4.16

OmphA assignments

Table S11: Chemical shift assignments for OmphA are provided for different solvents mixtures. Q atoms denote chemically equivalent protons in methyl groups.

atom			$CDCl_3$ / n-hexadecane-D_{34} C1 δ / ppm	$CDCl_3$ / n-hexadecane-D_{34} C2 δ / ppm	CD_3OD / H_2O δ / ppm
1	TRP	H	6.35	7.02	8.81
1	TRP	HA	5.23	5.28	5.23
1	TRP	HB2	3.32	3.18	3.11
1	TRP	HB3	3.22	3.23	3.25
1	TRP	HD1	7.15	7.15	7.14
1	TRP	HE1			10.33
1	TRP	HE3	7.64	7.84	7.66
1	TRP	HH2			7.05
1	TRP	HZ2			7.35
1	TRP	HZ3	7.16	7.17	7.12
2	MVA	HA	4.19	5.11	4.1
2	MVA	HB	2.18		2.12
2	MVA	QG2	0.49		0.39
2	MVA	QN	2.84	2.79	2.84
2	MVA	QG1			0.71
3	ILE	H	7.31	8.47	8.21
3	ILE	HA	4.8	4.73	4.65
3	ILE	HB	1.81	1.81	2.07
3	ILE	HG12	1		1.48
3	ILE	HG13			1.16
3	ILE	QD1			0.82
3	ILE	QG2	0.93		0.86
4	MVA	HA	5.09		5.18
4	MVA	HB	2.34		2.31
4	MVA	QG2	0.75	0.77	0.9
4	MVA	QN	3.01	3.1	3.1
4	MVA	QG1	0.94		0.75
5	MVA	HA	5.12		5.17

atom			$CDCl_3$ / n-hexadecane-D_{34} C1 δ / ppm	$CDCl_3$ / n-hexadecane-D_{34} C2 δ / ppm	CD_3OD / H_2O δ / ppm
5	MVA	HB	2.41		2.22
5	MVA	QG2	0.77		
5	MVA	QN	2.79	2.89	2.89
5	MVA	QG1	0.96	0.7	
6	SAR	HA2	5.03	4.92	4.82
6	SAR	HA3	3.3	3.2	3.69
6	SAR	QN	2.97		3.06
7	MVA	HA		5.29	5.07
7	MVA	HB		2.4	0.9
7	MVA	QG2			0.91
7	MVA	QN	2.92	2.92	2.9
7	MVA	QG1		0.99	0.81
8	IML	HA	5.24		5.1
8	IML	HB	2.3		2.32
8	IML	HD13	0.9		
8	IML	HG12			1.19
8	IML	QG1			1.29
8	IML	QG2			0.92
8	IML	QN	2.9	3	2.78
9	SAR	HA2	4.97	4.97	3.51
9	SAR	HA3	3.19	3.18	4.64
9	SAR	QN	3.09	3.09	3
10	VAL	H	7.12	6.84	8.21
10	VAL	HA	4.85	4.83	4.64
10	VAL	HB	2.2	2.23	1.98
10	VAL	QG1	0.8	1.07	0.98
10	VAL	QG2			0.91
11	IML	HA	5.31	5.32	5.17
11	IML	HB	2.15	2.15	2.07
11	IML	HD12	0.98		
11	IML	HG12	1.06		1.17
11	IML	HG23	0.9		
11	IML	QG1			2.07
11	IML	QN	3.05	3.08	3.11
12	SAR	HA2	4.14	3.87	3.53
12	SAR	HA3	3.88	4.3	4.45
12	SAR	QN	3.28	3.44	2.85

OmphA: distance restraints

Table S12: The distance restraints, UPLs (Upper Distance Limits) and LOLs (Lower Distance Limits), for OmphA in the three solvent mixtures are displayed. Table S5 shows the restraints for state 1 and state 2 specifically in the CD_3OH / H_2O mixture.

H_i			H_j			$CDCl_3$ / n-hexadecane-D_{34} C1 LOL / Å	UPL / Å	$CDCl_3$ / n-hexadecane-D_{34} C2 LOL / Å	UPL / Å	CD_3OH / H_2O LOL / Å	UPL / Å
1	TRP	H	1	TRP	HA	2.8	3.47				
1	TRP	H	1	TRP	HB2			1.95	4.55	1.8	4.14
1	TRP	H	1	TRP	HB3	3.09	3.48	3.17	3.61	1.8	4.14
1	TRP	H	1	TRP	QB					1.8	3.58
1	TRP	HA	1	TRP	HD1					2.03	4.73
1	TRP	HA	1	TRP	HE3	3.36	4.3	2.84	3.63		
1	TRP	HB3	1	TRP	HD1	2.38	5.56	2.08	4.84		
1	TRP	HE3	1	TRP	HZ3			2.27	2.37		
1	TRP	H	2	MVA	HA					1.8	5.02
1	TRP	H	2	MVA	QG2					1.8	5.5
1	TRP	HA	2	MVA	HA	2.11	2.35			1.8	2.92
1	TRP	HA	2	MVA	HB					1.8	5.15
1	TRP	HA	2	MVA	QG1					2.39	5.05
1	TRP	HA	2	MVA	QG2	3.09	4.17			1.97	4.16
1	TRP	HA	2	MVA	QN			2.31	2.93	2.4	5.06
1	TRP	HA	2	MVA	QG1	1.8	3.86	1.8	4.82		
1	TRP	HB2	2	MVA	HA					1.8	4.49
1	TRP	HB2	2	MVA	QG2					1.8	4.88
1	TRP	HB3	2	MVA	HA					1.8	4.49
1	TRP	HB3	2	MVA	QG2	3.25	4.24			1.8	4.88
1	TRP	HD1	2	MVA	HA					1.8	5.43
1	TRP	HD1	2	MVA	QG2					1.8	4.57
1	TRP	HE1	2	MVA	QN					2.6	5.49
1	TRP	QB	2	MVA	HA					1.8	3.92
1	TRP	QB	2	MVA	QG2					1.8	4
1	TRP	QB	2	MVA	QG1	1.8	4.82				
1	TRP	H	3	ILE	QD1					1.8	5.26
1	TRP	HA	3	ILE	H	1.8	5.5			1.4	3.28
1	TRP	HA	3	ILE	HB					1.8	5.5
1	TRP	H	11	IML	QN	2.91	3.45				
1	TRP	H	12	SAR	HA2	2.94	3.15	2.52	2.94	1.8	4.01
1	TRP	H	12	SAR	HA3	1.8	4.37	3.06	3.21	1.8	4.01
1	TRP	H	12	SAR	QA	1.8	3.79	1.8	3.7		

H_i			H_j			$CDCl_3$ / n-hexadecane-D_{34} C1 LOL / Å	UPL / Å	$CDCl_3$ / n-hexadecane-D_{34} C2 LOL / Å	UPL / Å	CD_3OH / H_2O LOL / Å	UPL / Å
1	TRP	H	12	SAR	QN	1.8	5.5			1.8	4.47
1	TRP	QB	12	SAR	QA					1.8	4.94
2	MVA	HA	2	MVA	QG1					1.92	2.46
2	MVA	HA	2	MVA	QG2					1.97	2.59
2	MVA	HA	2	MVA	QN					1.8	3.55
2	MVA	HA	2	MVA	QG1	1.8	3.65	1.8	4.12		
2	MVA	HB	2	MVA	QN	2.28	3.28			1.85	2.68
2	MVA	QG1	2	MVA	QN					2.28	3.64
2	MVA	QG2	2	MVA	QN	2.32	3.64			1.94	3.03
2	MVA	QN	2	MVA	QG1	1.8	3.92				
2	MVA	HA	3	ILE	H	1.6	3.74			1.8	3.2
2	MVA	HA	3	ILE	HB					1.8	5.32
2	MVA	QG1	3	ILE	H					2.47	3.39
2	MVA	QG2	3	ILE	H					2.81	4.13
2	MVA	QN	3	ILE	H	1.8	4			1.8	4.55
2	MVA	QN	3	ILE	HA					2.35	4.95
2	MVA	QN	3	ILE	QG1	1.8	5.5				
2	MVA	QN	3	ILE	QG2	1.8	5.5				
2	MVA	HA	5	MVA	QN			2.3	2.9		
3	ILE	H	3	ILE	HA			2.19	5.11		
3	ILE	H	3	ILE	HB					2.08	2.58
3	ILE	H	3	ILE	QD1					1.62	3.11
3	ILE	H	3	ILE	QG1					1.8	4.15
3	ILE	H	3	ILE	QG2					2.55	4.17
3	ILE	HA	3	ILE	HB	2.89	3.46	3.14	3.55		
3	ILE	HA	3	ILE	HG12	1.94	4.54				
3	ILE	HA	3	ILE	QD1					1.8	3.91
3	ILE	HA	3	ILE	QG2	1.96	4.12			2.16	2.93
3	ILE	H	4	MVA	QN					1.8	4.62
3	ILE	H	4	MVA	QG1					1.8	4.78
3	ILE	HA	4	MVA	QN	2.07	2.79	2.16	2.76	1.68	2
3	ILE	HA	4	MVA	QG1					1.8	4.23
3	ILE	HB	4	MVA	QN	2.37	5	2.77	3.99		
3	ILE	QG1	4	MVA	QN					1.8	4.25
3	ILE	HA	5	MVA	QG1			1.8	5.38		
4	MVA	HA	4	MVA	QG2	2.02	4.26	1.41	2.97		
4	MVA	HA	4	MVA	QN	2.26	2.73			1.9	4.01
4	MVA	HA	4	MVA	QG1	2.1	4.43	1.63	3.44	1.8	2.98
4	MVA	HB	4	MVA	QG2	1.94	4.1				
4	MVA	HB	4	MVA	QN					1.86	2.66
4	MVA	HB	4	MVA	QG1	2	4.23				

H_i			H_j			$CDCl_3$ / n-hexadecane-D_{34} C1 LOL / Å	UPL / Å	$CDCl_3$ / n-hexadecane-D_{34} C2 LOL / Å	UPL / Å	CD_3OH / H_2O LOL / Å	UPL / Å
4	MVA	QG2	4	MVA	QN			2.38	4.48		
4	MVA	QN	4	MVA	QG1	1.8	3.68	1.8	3.65	1.8	2.67
4	MVA	HA	5	MVA	QN	1.8	3.53	1.8	3.01	1.46	3.09
4	MVA	HB	5	MVA	QN	2.23	2.9				
4	MVA	QG2	5	MVA	QN	2.47	4.71				
4	MVA	QN	5	MVA	HB	2.52	3.42				
4	MVA	QN	5	MVA	QN					2.16	3.19
4	MVA	QN	5	MVA	QG1			1.59	3.79		
4	MVA	QQG1	5	MVA	QN	2.7	5.15			1.8	3.79
4	MVA	HA	7	MVA	HB			1.39	3.25		
4	MVA	QN	7	MVA	HB			1.91	3.12		
5	MVA	HA	5	MVA	HB	3.09	3.32				
5	MVA	HA	5	MVA	QN	2.15	2.92				
5	MVA	HA	5	MVA	QG1	1.8	4.06				
5	MVA	HB	5	MVA	QN	1.8	3.76			2.02	2.52
5	MVA	QN	5	MVA	QG1	1.8	4.67	1.8	4.35		
5	MVA	HA	6	SAR	QA	1.8	4.71				
5	MVA	HA	6	SAR	QN	2.1	2.81			1.43	3.02
5	MVA	QN	6	SAR	QA	1.8	4.19				
5	MVA	QN	6	SAR	QN					1.8	4.61
5	MVA	QQG1	6	SAR	QA	1.8	4.06				
5	MVA	QQG1	6	SAR	QN	1.8	4.1				
6	SAR	HA2	6	SAR	QN	2.71	3.35			1.8	3.55
6	SAR	HA3	6	SAR	QN	2.12	2.68			1.8	3.55
6	SAR	QA	6	SAR	QN	1.8	3.15			1.8	2.84
6	SAR	HA2	7	MVA	QN	2.26	2.76	2.57	3.05	1.64	3.45
6	SAR	HA3	7	MVA	QN	2.21	2.64			1.72	3.62
6	SAR	QA	7	MVA	HB					1.8	5.34
6	SAR	QA	7	MVA	QN	1.8	3.17	1.8	3.5	1.8	3.55
6	SAR	QA	7	MVA	QG1					1.8	3.97
6	SAR	QN	7	MVA	QN					1.8	4.88
7	MVA	HA	7	MVA	QG1					1.06	2.23
7	MVA	HA	7	MVA	QG2					1.8	4.13
7	MVA	HA	7	MVA	QN			2.51	5.3	2.16	4.56
7	MVA	HA	7	MVA	QG1			2.1	4.43		
7	MVA	HA	8	IML	QN			2.27	2.78	1.8	2.77
7	MVA	QN	8	IML	QG1					2	3.71
7	MVA	QN	8	IML	QN					1.7	2.43
8	IML	HA	8	IML	HG22	1.8	4.13				
8	IML	HA	8	IML	QG1	2.47	5.21				
8	IML	HA	8	IML	QG2	2.05	4.33				

H_i			H_j			$CDCl_3$ / n-hexadecane-D_{34} C1 LOL / Å	UPL / Å	$CDCl_3$ / n-hexadecane-D_{34} C2 LOL / Å	UPL / Å	CD_3OH / H_2O LOL / Å	UPL / Å
8	IML	HB	8	IML	QG1	1.9	4				
8	IML	HB	8	IML	QN	2.58	3.1			2.32	4.88
8	IML	HG22	8	IML	QN	1.8	4.46				
8	IML	QD1	8	IML	QN	3.03	5.78				
8	IML	QG1	8	IML	QN					1.8	3.7
8	IML	QG2	8	IML	QN					1.8	2.89
8	IML	HA	9	SAR	QN	2.38	3.05	1.32	2.78		
8	IML	HB	9	SAR	QN	1.8	4.04				
8	IML	HD13	9	SAR	QN	1.8	5.5				
8	IML	HG22	9	SAR	QN	1.8	5.5				
8	IML	QD1	9	SAR	QN	2.91	5.55				
8	IML	QG1	9	SAR	QN					1.8	4.45
8	IML	QN	9	SAR	QN					1.8	3.85
9	SAR	HA2	9	SAR	QN					1.8	3.38
9	SAR	HA3	9	SAR	QN					1.8	3.38
9	SAR	QA	9	SAR	QN	1.8	2.53				
10	VAL	H	8	IML	QN	1.8	5.5	1.8	5.36		
10	VAL	QQG	8	IML	QN			1.8	5.09		
10	VAL	H	9	SAR	HA2	2.92	3.23			2.36	2.69
10	VAL	H	9	SAR	HA3	1.97	4.61			1.8	3.47
10	VAL	H	9	SAR	QN			1.8	4.68	2.98	3.65
10	VAL	QQG	9	SAR	QA					1.8	4.43
10	VAL	H	10	VAL	HA			2.88	3.27		
10	VAL	H	10	VAL	HB					2.43	3.55
10	VAL	H	10	VAL	QG1					1.8	4.1
10	VAL	H	10	VAL	QG2					1.8	4.1
10	VAL	H	10	VAL	QG	1.8	4.12				
10	VAL	HA	10	VAL	HB	2.91	3.81	2.8	3.51		
10	VAL	HA	10	VAL	QG1			2.05	4.33	1.8	3.39
10	VAL	HA	10	VAL	QG2			2.46	5.19	1.8	3.39
10	VAL	H	11	IML	QN			1.8	4.98	1.8	4.86
10	VAL	HA	11	IML	QG1	2.08	4.38				
10	VAL	HA	11	IML	QN	2.11	2.52	2.07	2.65	1.39	2.94
10	VAL	HB	11	IML	QG1	2.47	5.2				
10	VAL	HB	11	IML	QN	1.8	4.07	2.51	3.6	2.54	4.4
10	VAL	QG1	11	IML	HA			2.38	5.02		
10	VAL	QQG	11	IML	QN					1.8	3.51
11	IML	QG2	5	MVA	HA	2.32	4.88				
11	IML	HA	11	IML	HG12	1.92	4.48				
11	IML	HA	11	IML	HG23	1.8	3.14				
11	IML	HA	11	IML	QG1	1.8	3.64	1.8	3.69		

H_i			H_j			$CDCl_3$ / n-hexadecane-D_{34} C1 LOL / Å	UPL / Å	$CDCl_3$ / n-hexadecane-D_{34} C2 LOL / Å	UPL / Å	CD_3OH / H_2O LOL / Å	UPL / Å
11	IML	HA	11	IML	QG2	1.85	3.91				
11	IML	HA	11	IML	QN			1.8	3.77		
11	IML	HB	11	IML	HD12	1.8	3.11				
11	IML	HB	11	IML	QG2	2.21	4.66				
11	IML	HB	11	IML	QN	2.37	3.35	2.3	4.85	1.8	4
11	IML	QG1	11	IML	QN	1.8	4.24			1.8	4.22
11	IML	HA	12	SAR	QN	2.1	2.91	1.99	3.06		
12	SAR	QA	3	ILE	H					1.8	5.31
12	SAR	QA	3	ILE	HB					1.8	5.26
12	SAR	QA	3	ILE	QD1					1.8	4.55
12	SAR	HA3	5	MVA	HA	3.9	3.9				
12	SAR	QA	5	MVA	QN			1.8	5.34		
12	SAR	QN	5	MVA	HA	2.8	3.83				
12	SAR	QN	5	MVA	QN	2.3	3.38	1.8	4.12		
12	SAR	HA2	12	SAR	QN	2.62	3.3	1.8	4.27	1.8	3.52
12	SAR	HA3	12	SAR	QN	2.48	3.15	2.35	3.14	1.8	3.52
12	SAR	QA	12	SAR	QN	1.8	3.3			1.8	3.05

References

(1) Widmer, A. Witnotp: A Computer Program for Molecular Modeling. Novartis: Basel 1997.

(2) Clark, M.; Cramer, R. D.; Van Opdenbosch, N. Validation of the General Purpose Tripos 5.2 Force Field. *J. Comput. Chem.* **1989**, *10* (8), 982–1012. https://doi.org/10.1002/jcc.540100804.

(3) Sterner, O.; Etzel, W.; Mayer, A.; Anke, H. Omphalotin, a New Cyclic Peptide with Potent Nematicidal Activity from Omphalotus Olearius. II. Isolation and Structure Determination. *Nat. Prod. Lett.* **1997**, *10* (1), 33–38. https://doi.org/10.1080/10575639708043692.

(4) Güntert, P.; Buchner, L. Combined Automated NOE Assignment and Structure Calculation with CYANA. *J. Biomol. NMR* **2015**, *62* (4), 453–471. https://doi.org/10.1007/s10858-015-9924-9.

(5) Strotz, D.; Orts, J.; Chi, C. N.; Riek, R.; Vögeli, B. ENORA2 Exact NOE Analysis Program. *J. Chem. Theory Comput.* **2017**, *13* (9), 4336–4346. https://doi.org/10.1021/acs.jctc.7b00436.

(6) Van Rossum, G.; Drake, F. L. *Python 3 Reference Manual*; CreateSpace: Scotts Valley, CA, 2009.

(7) Hu, H.; Krishnamurthy, K. Revisiting the Initial Rate Approximation in Kinetic NOE Measurements. *J. Magn. Reson.* **2006**, *182* (1), 173–177. https://doi.org/10.1016/j.jmr.2006.06.009.

(8) Pedregosa, F.; Varoquaux, G.; Gramfort, A.; Michel, V.; Thirion, B.; Grisel, O.; Blondel, M.; Prettenhofer, P.; Weiss, R.; Dubourg, V.; Vanderplas, J.; Passos, A.; Cournapeau, D.; Brucher, M.; Perrot, M.; Duchesnay, E. Scikit-Learn: Machine Learning in {P}ython. *J. Mach. Learn. Res.* **2011**, *12*, 2825–2830.

(9) Xuan Vinh, N.; Epps, J.; Bailey, J. Information Theoretic Measures for Clusterings Comparison: Variants, Properties, Normalization and Correction for Chance. *J. Mach. Learn. Res.* **2010**, *11*, 2837–2854.

(10) Virtanen, P.; Gommers, R.; Oliphant, T. E.; Haberland, M.; Reddy, T.; Cournapeau, D.; Burovski, E.; Peterson, P.; Weckesser, W.; Bright, J.; van der Walt, S. J.; Brett, M.; Wilson, J.; Millman, K. J.; Mayorov, N.; Nelson, A. R. J.; Jones, E.; Kern, R.; Larson, E.; Carey, C. J.; Polat, Ii.; Feng, Y.; Moore, E. W.; VanderPlas, J.; Laxalde, D.; Perktold, J.; Cimrman, R.; Henriksen, I.; Quintero, E. A.; Harris, C. R.; Archibald, A. M.; Ribeiro, A. H.; Pedregosa, F.; van Mulbregt, P.; SciPy 1.0 Contributors. {SciPy} 1.0: Fundamental Algorithms for Scientific Computing in Python. *Nat. Methods* **2020**, *17*, 261–272. https://doi.org/10.1038/s41592-019-0686-2.

(11) Kessler, H.; Köck, M.; Wein, T.; Gehrke, M. Reinvestigation of the Conformation of Cyclosporin A in Chloroform. *Helv. Chim. Acta* **1990**, *73* (7), 1818–1832. https://doi.org/10.1002/hlca.19900730703.

(12) Case, D. A.; Aktulga, H. M.; Belfon, K.; Ben-Shalom, I. Y.; Berryman, J. T.; Brozell, S. R.; Cerutti, D. S.; Cheatham, T. E. I.; Cisneros, G. A.; Cruzeiro, V. W. D.; Darden, T. A.; Duke, R. E.; Giambasu, G.; Gilson, M. K.; Gohlke, H.; Goetz, A. W.; Harris, R.; Izadi, S.; Izmailov, S.

A.; Kasavajhala, K.; Kaymak, M. C.; King, E.; Kovalenko, A.; Kurtzman, T.; Lee, T. S.; LeGrand, S.; Li, P.; Lin, C.; Liu, J.; Luchko, T.; Luo, R.; Machado, M.; Man, V.; Manathunga, M.; Merz, K. M.; Miao, Y.; Mikhailovskii, O.; Monard, G.; Nguyen, H.; O'Hearn, K. A.; Onufriev, A.; Pan, F.; Pantano, S.; Qi, R.; Rahnamoun, A.; Roe, D. R.; Roitberg, A.; Sagui, C.; Schott-Verdugo, S.; Shajan, A.; Shen, J.; Simmerling, C. L.; Skrynnikov, N. R.; Smith, J.; Swails, J.; Walker, R. C.; Wang, J.; Wang, J.; Wei, H.; Wolf, R. M.; Wu, X.; Xiong, Y.; Xue, Y.; York, D. M.; Zhao, S.; Kollman, P. A. Amber22 and AmberTools22. University of California, San Francisco. 2022.
